# Serial cycle threshold to assess the infectious potential of SARS-CoV-2: A systematic review

**DOI:** 10.1017/S0950268826101484

**Published:** 2026-05-06

**Authors:** Elena Cecilia Rosca, Jason Oke, Tom Jefferson, Jon Brassey, Igho Onakpoya, Annette Plüddemann, Sara Gandini, Susanna Maltoni, David Evans, John Conly, Carl Heneghan

**Affiliations:** 1 Victor Babes University of Medicine and Pharmacy, Timisoara, Romania; 2 Centre for Evidence-Based Medicine, University of Oxford, UK; 3 Department for Continuing Education, University of Oxford, UK; 4 Trip Database Ltd, Newport, UK; 5 Department of Experimental Oncology, IEO European Institute of Oncology IRCCS, 20141 Milan, Italy; 6 Clinical Trial Centre Unit, IRCCS Azienda Ospedaliero-Universitaria di Bologna, 40138 Bologna, Italy; 7 Li Ka Shing Institute of Virology and Dept. of Medical Microbiology & Immunology, University of Alberta, Canada; 8 Departments of Medicine, Microbiology, Immunology & Infectious Diseases, and Pathology & Laboratory Medicine, Synder Institute for Chronic Diseases and O’Brien Institute for Public Health, Cumming School of Medicine, University of Calgary and Alberta Health Services, Calgary, Canada

**Keywords:** COVID-19, cycle threshold, SARS-CoV-2, transmission, infectiousness, monitoring

## Abstract

We sought to assess predictive factors for SARS-CoV-2 infectiousness using a meta-analytic approach. We searched LitCovid, medRxiv, Google Scholar, and the WHO COVID-19 database until June 30 2025, including studies which cultured SARS-CoV-2, relating them to clinico-epidemiologic and laboratory variables and RT-PCR cycle threshold (Ct) values. Using linear mixed-effects regression models, we tested for independent associations with Ct values with 95%CIs and adjusted *P*-values in a multivariable model. We used a modified QUADAS criteria to assess risk-of-bias. We included 50 studies, with 39 in quantitative synthesis. The percentage of culture-positive specimens decreased with increasing Ct values (subgroup test difference Q = 96.71;*P* < 0.001) and time since the first PCR test (Q = 26.95;*P* = 0.0026). Presence of symptoms (Q = 20.1;*P* < 0.01), gene platform used (Q = 14.89;*P* = 0.002), being a cancer patient (Q = 24.9;*P* < 0.0001), and vaccination status (Q = 8.80;*P* = 0.012) were associated with increased culture-positivity, whereas a rising Ct (adjusted Ct change −6.58[95%CI] -5.30, −7.86;*P* < 0.001) was strongly associated with culture-negativity. Analysing 186 immunocompetent patients with 1,393 Ct values, 2 consecutive Cts ≥ 30 or a rising Ct value on serial testing demonstrated a sensitivity of 87.5% and specificity of 96.3% using culture positivity as the outcome. Serial Ct monitoring, integrated with clinico-epidemiologic data is a valuable tool for assessing infectiousness, providing objective criteria for discontinuing isolation and guiding clinical decisions.

## Introduction

Effective prevention and management of SARS-CoV-2 infections relies on identifying those who are infected or potentially infectious. The most commonly used method for detection is reverse transcriptase quantitative polymerase chain reaction (RT-qPCR) [1–3]. Amplification of genomic sequences occurs in a sample mixture with repeated thermal cycles adding copies of the target nucleic acids, usually measured in cycle thresholds (Ct). The lower the target nucleic acid amount in a sample, the longer it takes to reach a critical ‘threshold’ above baseline based on fluorescent signal detection. The Ct is ‘the thermal cycle number at which the fluorescent signal exceeds that of the background and thus passes the threshold for positivity’ [2].

Transmission from one host to another requires replication-competent SARS-CoV-2 to allow attachment to cellular targets and subsequent invasion. Specimens with a high Ct are unlikely to be culturable and have no potential for SARS-CoV-2 transmission [3]. There is a relationship between time of symptom onset, test timing, Ct, and symptom severity in immunocompetent hosts. Qualitative PCR cannot detect infectiousness, requiring integration and interpretation with medical, drug and exposure information, and using probes and methods validated against viral culture and gene sequencing. Few studies report these variables [4, 5].

Non-standardized reporting and testing has created interpretation challenges. For example, Ct reporting can be inconsistent, with differing platforms and testing methodologies, while cultures are expensive, laborious, time-consuming, and cannot be done outside Level 3 laboratories. We explored whether serial Ct measurements from the same individuals, using the same platform in the same laboratory with appropriate internal controls, could be substituted for viral cultures to facilitate decision-making regarding infectiousness. A declining Ct value with or without symptoms could signify an increased viral load and the possibility of becoming or being infectious [3], while a reverse situation could facilitate the decision to remove isolation precautions.

In our previous review, we included 29 studies (with searches until 10 September 2020) reporting attempts at culturing or observing tissue infection by SARS-CoV-2 in different specimens [3]. We sought to update the searches to explore the possibility of constructing a predictive comparative cohort integrating PCR and viral culture results from respiratory and other clinical specimens and detailed anamnestic, clinical, and epidemiologic data.

## Methods

This update addressed the following questions:

1. What is the relationship between symptom onset, disease course, infectious status, immunologic status, vaccination, and changes in serial Ct values of SARS-CoV-2?
2. What is the relationship between the type of gene platform used and the change in Ct values of SARS-CoV-2?
3. Is there a single and/or complex set of variables that makes the association of a Ct more or less likely predictive of culturability and thus infectiousness?

### Search

We updated our searches using LitCovid, medRxiv, Google Scholar, and the WHO Covid-19 database. The search terms used for identifying appropriate articles were CPE OR ‘cytopathic effect’ OR ‘Viral culture’ OR ‘virus culture’ OR ‘vero’ OR ‘virus replication’ OR ‘viral replication’ OR ‘cell culture’ OR ‘viral load’ to 30 June 2025. For medRxiv and Google Scholar, the terms coronavirus OR covid-19 OR SARS-CoV-2 were used to identify COVID-19 related articles. An information specialist [JB] undertook searches. Citation matching, forwards and backwards, were undertaken for key relevant articles. Only English-language articles were included. Two reviewers independently screened search results; a third adjudicator was used when required.

### Inclusion and exclusion criteria

We included studies reporting attempts to culture SARS-CoV-2 and related them to exposure date, symptom onset to test, and detailed characteristics of the individual tested. We included adult persons exposed to SARS-CoV-2 in any setting who had undergone more than one RT-PCR assay during an acute respiratory episode of SARS-CoV-2 infection providing one or more respiratory or oropharyngeal specimens or other relevant environmental samples in their proximity.

We defined culture as encompassing several methods detecting exponential virus growth in cell culture and confirmed the replicating agent as SARS-CoV-2 (cell culture or live animal inoculation with verification techniques [PCR, immunological staining, or RNA gene sequencing]). Isothermal detection methods were not included.

We assessed observational cohort and case series studies with attempts to culture SARS-CoV-2. A successful comparison between the results of viral culture and surrogate methods, including rapid antigen tests, serial PCR trajectories, measuring Ct cut-offs, PCR targeting sub-genomic RNAs, or quantitative PCR (droplet digital PCR or internally standardized PCR) would support the use of such tests in identifying infectious cases. Identification of such individuals and the likelihood of infectivity would achieve objective 3. We excluded studies reporting aggregate Ct data or graphically displayed Ct values without individual results being available.

### Data extraction

One reviewer extracted data, while a second reviewer checked the extractions. We extracted data from individuals tested on PCR positive/total number tested, with as many repeat values over time as possible; details of PCR methodology including use of internal controls, Ct values over time, date of symptom onset, symptoms, and veracity of symptom ascertainment (where available), date of symptom resolution, viral culture results/total number tested, details of viral culture methods, and SARS-CoV-2 identification.

### Analysis

We report the number of observations, time from first RT-PCR, and symptom status.

We performed subgroup analyses by platform type, comorbidities, immune status, hospitalization, use of treatments, including immunotherapy, and SARS-CoV-2 lineage.

We tested the hypothesis that there is an association between positive growth of SARS-CoV-2 from cell culture and the first test result for RT-PCR Ct. We examined variables that may influence the interpretation of the Ct: gene, demographics, comorbidities, and baseline laboratory measures within 24 h of specimen collection. We also tested a secondary hypothesis that a trending analysis of serial Cts combined with clinical data from non-immunocompromised COVID-19 patients could reliably predict culture positivity and hence non-infectiousness based on reliable SARS-CoV-2 cell culture techniques done using internal controls, which could be used as a surrogate by clinicians for discontinuation of isolation.

To quantify how exogenous factors may influence Ct values, we fitted linear mixed-effects models with the first Ct value per individual as the outcome. Predictor variables were included as fixed effects, while the study was included as a random intercept to account for clustering of observations across studies. Univariate models were first fitted for each predictor separately. Predictors were prespecified based on clinical relevance and univariate associations (*P* < 0.10; see Table 1 and Supplementary WebTable 4). Forward selection (entry *P* < 0.05, removal *P* > 0.10) was then applied in the multivariable linear mixed-effects model to identify factors independently associated with Ct values. Results are reported as changes in Ct values with 95% confidence intervals (CIs) and adjusted *P*-values. All analyses were performed in R using the meta and lme4 packages.

**Figure 1.**
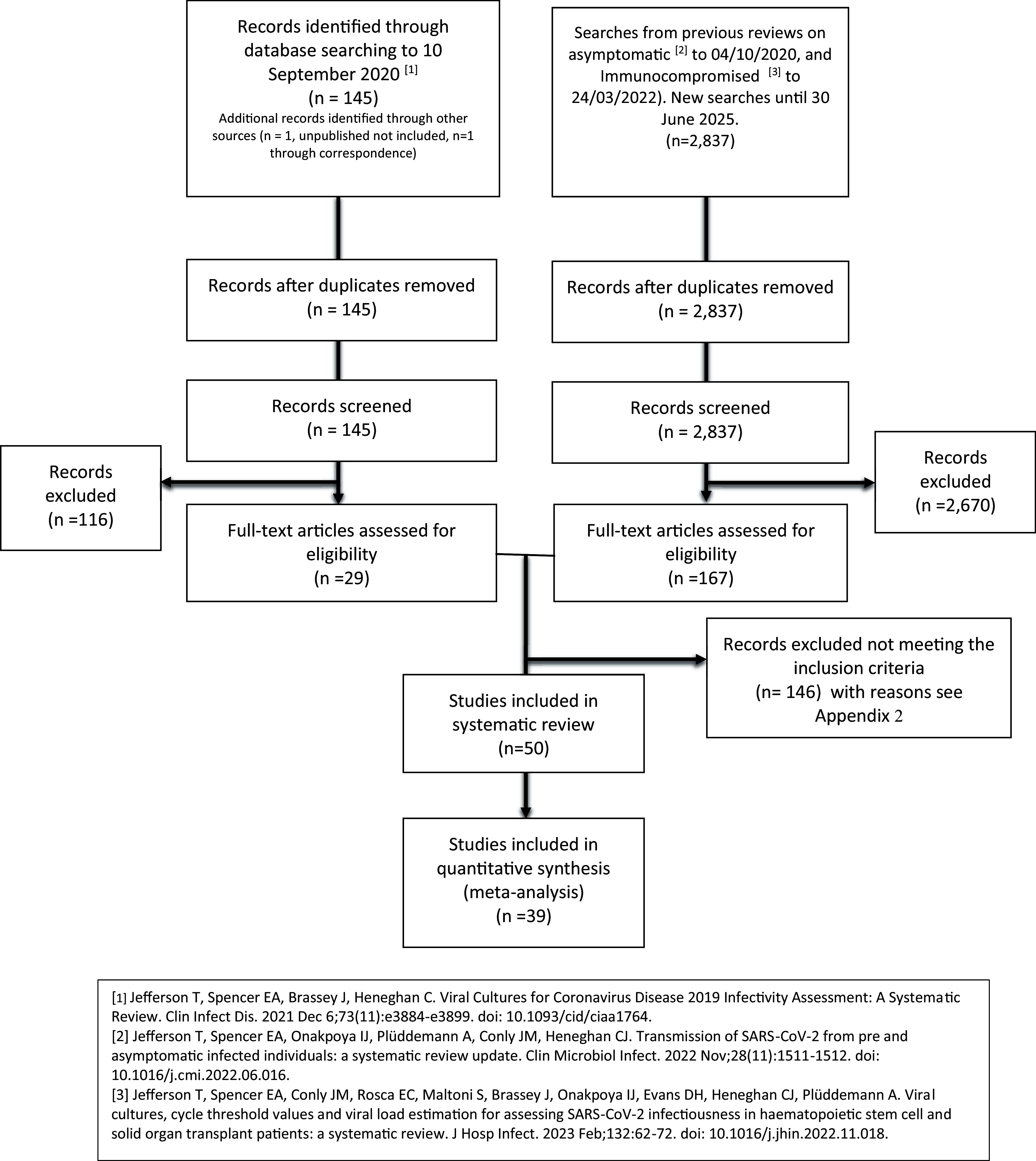
Flow chart. Figure 1. long description.

We used the same approach to test independent associations in a multivariable model and applied a forward selection method to isolate factors independently associated with the Ct value. We report the univariate associations with changes in Ct values and 95% CIs and multivariate independent associations with Ct values with 95% CIs and adjusted *P*-values. All analyses were performed in-house using R and the meta and lme4 packages [6, 7]. The descriptive trending analysis for Ct values in immunocompetent patients was done using Python 3.13.5 (containing Pandas 2.3.0).

We assessed associations between culture positivity as the main outcome and key clinical and demographic factors (predictors) using a two-stage meta-analysis approach. For each factor, we pooled study-level estimates of the positivity rate stratified by subgroups. We used inverse-variance weighting and logit transformed proportions. Percentage culture positivity with 95% CIs at the subgroup level were estimated and differences in positivity rates between subgroups tested.

### Quality assessment

We assessed the quality of the included studies according to modified QUADAS criteria that we have described previously [3, 8] (Supplementary Appendix 1). We followed PRISMA reporting guidelines [9]. This protocol arises out of our previously published protocol (https://osf.io/5dy6e) [10].

## Results

We included searches from previous reviews from the date of our last review search, 20 September 2020 until 30 June 2025. We screened a further 2,837 articles from the 145 already screened in September 2020; we reviewed 196 full-text articles for inclusion. Three reviewers (ES, CR, and TJ) independently assessed all the screened articles for inclusion (Figure 1). After a full-text review, we excluded 146 (Supplementary Appendix 2) and included 50 studies in the review and 39 in the quantitative synthesis (Supplementary Appendix 3).

**Figure 2.**
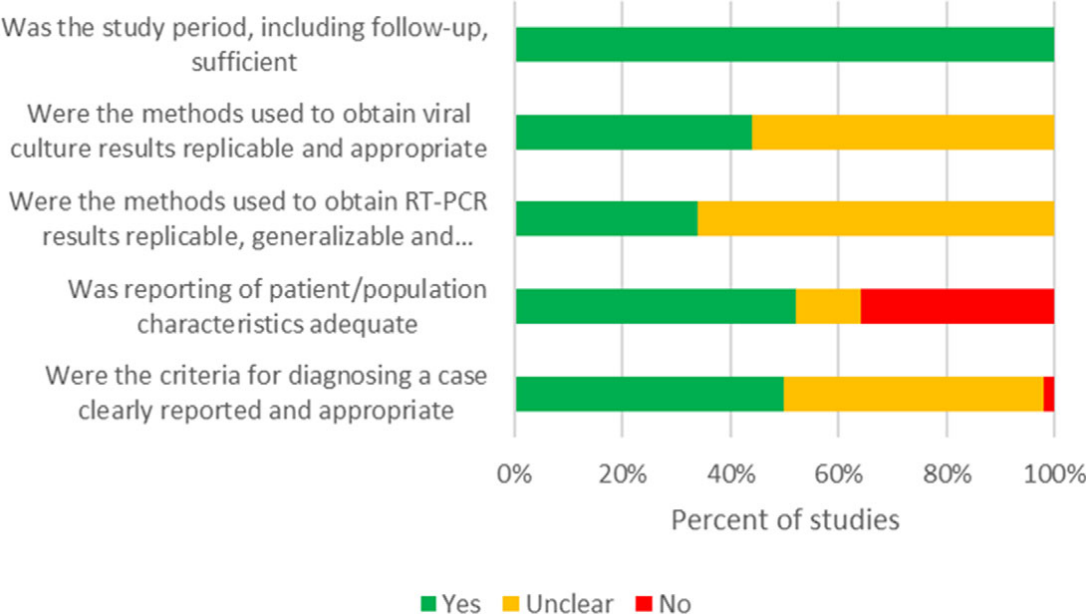
Risk of bias assessment. Figure 2. long description.

We excluded 11 studies from the analysis because they did not report individual RT-PCR Ct values. Specifically, three studies provided viral culture data without corresponding Ct values [11–13] and eight reported viral load as log10 copies instead [14–21]. Seven patients from the quantitative synthesis group were excluded because their culture status and Ct value did not align (i.e. not taken simultaneously).

Table 2 shows the characteristics of each study. We extracted data from 619 individuals, with the number of individuals included in each study ranging from 1 to 82. Sixteen studies were conducted in the USA; five in Italy; four in Canada; three each in Austria, Brazil, Korea, and Germany; two studies each in Denmark, Japan, and the UK; and one each in China, France, Israel, Portugal, Saudi Arabia, Spain, and Sweden (Table 2). We included data from 3,745 samples across 50 studies. Supplementary WebTable 1 reports the symptoms, the veracity of symptoms and signs checking, and the associated medical history of the study participants. Supplementary WebTable 2 reports the PCR sample collection methods, management, platform used, culture methods, cell lines, and controls.

We could not identify a protocol for any study. The 50 studies included were all case series and the overall risk of bias was moderate (Table 3, Figure 2).

**Figure 3.**
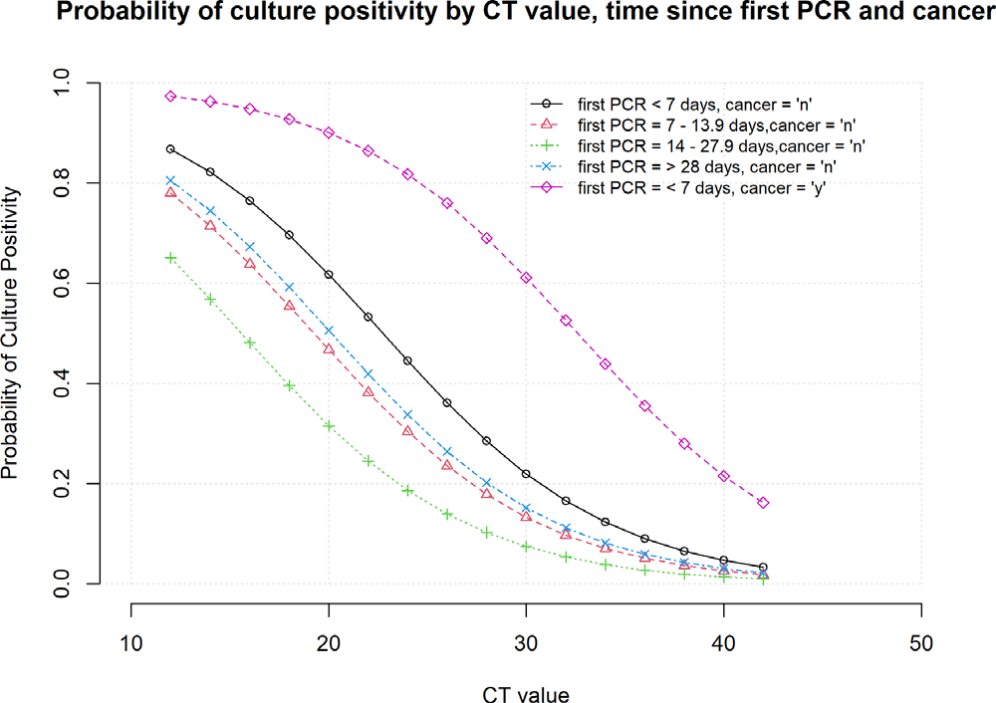
Probability of culture positivity by Ct value, time since first RT-PCR and cancer. Figure 3. long description.

### Relationship between symptom onset, disease course, infectious status, immunologic status, vaccination, and change in Ct values of SARS-CoV-2

Table 4 reports the first Ct test result. We included 39 studies and 381 patients with a median of 3 (IQR 2–7) observations (per individual) and a median of 5 days (IQR 0–11) from the first RT-PCR test following the first result. The number of individuals who were culture positive at the first test was 148/381 (38.8%), and the number of symptomatic patients was 253/381 (66.4%). A descriptive analysis of serial Cts from non-immunocompromised COVID-19 patients only is shown in Supplementary WebTable 3 (n = 11 studies; n = 186 patients; n = 1,393 Ct values) and revealed a median and interquartile range (IQR) Ct value for positive (336) vs. negative (1057) viral cultures of 19.5 and 6.1 vs. 32.8 and 11.0, respectively. The mean Ct values for samples with positive (20.5) and negative (32.7) cultures were consistent with the median values. An analysis of patients assessing culture positivity as the outcome and sample Ct values <30 and > 2 or more Ct values and with 2 consecutive Cts ≥ 30 or a rising Ct value on serial testing with one value ≥30, demonstrated a sensitivity of 87.5%, a specificity of 96.3%, a positive predictive value of 88.2% and a negative predictive value of 96.0% with predictive values calculated using the observed prevalence of viral culture positivity within the serial Ct cohort of 24.1% in 186 patients. There were a number of outliers where cultures were reported as positive for viral growth despite a Ct ≥ 30 and all either used repetitive subculturing to obtain growth or were a single reported positive growth amidst multiple numbers of repeated negative cultures for the same patient. An analysis of outliers for positive viral cultures with a Ct ≥ 30 using the 1.5xIQR rule found that 14 outliers with a Ct above the upper bound of 32.1 confirmed the latter finding as well. Cultures that were negative for viral growth despite a Ct value of <25.6 (median + IQR for positive cultures) may be due to poor collection techniques, improper handling, freeze–thaw cycles, faulty inoculation, cell line choice, and contamination, all of which are well recognized in cell culture techniques [22]. Review of the original reports indicated that these outlier observations originated from four studies and often occurred later in infection or required multiple serial culture passages until viral growth was observed or were a single culture-positive sample sandwiched between multiple prior negative cultures and followed by subsequent negative cultures. The choice of a Ct value of ≥30 as a threshold choice was based on several studies using multiple primer sets and cell culture techniques, including quantitative cultures in one study, revealing it to be a consistent cut-off for demonstrating non culturable virus on cell culture [5, 23, 24], and some of the authors corroborated their findings using ROC curves.

Table 1 reports the overall culture-positivity results using a two-stage meta-analysis approach that demonstrated the percentage of culture-positivity decreases with increased Ct value (test for subgroup difference Q = 96.71, *P* < 0.001), and with time since the first PCR test (Q = 26.95, *P* = 0.0026). The presence of symptoms (Q = 20.19, *P* < 0.01) and vaccination status (Q = 8.80, *P* = 0.012) increased the proportion of culture-positive results, as did the type of gene platform used (Q = 14.89, *P* = 0.002). We found a significant interaction for cancer patients (Q = 24.9, *P* < 0.0001) and patients on COVID-19 treatments such as remdesivir, dexamethasone, and others (Q = 7.86, *P* = 0.020). There were no subgroup interactions for hospitalization, intensive care admission, or the presence of at least one chronic condition.

### Independent associations with the cycle threshold value

Supplementary WebTable 4 reports univariate associations with a Ct value based on first test values per individual. Table 5 reports the independent associations with Ct values. Culture positivity was associated with a reduction in the Ct value (adjusted change in Ct −6.58, 95% CI, −5.30, −7.86, *P* < 0.001); time since the first PCR test was independently associated with a Ct increase (*P* < 0.001); the use of COVID-19 treatments led to Ct reduction (adjusted change in Ct −5.61, 95%CI, −8.78, −2.43, *P* < 0.001). Table 6 reports multivariate analysis on independent associations with culture positivity. The probability of culture positivity by Ct value, time since first RT-PCR and cancer are presented in Figure 3. The probability of positive cultures was more likely with low Cts.

**Table 1.** Culture positivity (using a two-stage meta-analysis approach) Table 1. long description.

Characteristic	Number of studies (number of patients)	Percentage culture positive (95% CI) (reference level)	Q	I2
**CT value**				
< 20	k = 22 (97)	74.2% (64.6 to 81.9)	12.4	0.0%
20–24	k = 20 (84)	64.3% (53.5 to 73.8)	11.2	0.0%
25–29	k = 17 (62)	24.2% (15.1 to 36.3)	2.38	0.0%
30–34	k = 15 (71)	7.04% (2.96 to 15.8)	0.00	0.0%
35 +	k = 12 (67)	2.99% (0.75 to 11.2)	0.00	0.0%
Test for subgroup differences	Q = 96.71, df = 4		*P* < 0.001	
**Time since first PCR**				
< 7 days	k = 17 (n = 183)	51.9% (44.7 to 59.1)	22.47	28.2%
7–13 days	k = 16 (67)	34.3% (24.0 to 46.4)	4.71	0.0%
14–27 days	k = 16 (99)	21.2% (14.3 to 30.4)	1.43	0.0%
+28 days	k = 16 (32)	28.1% (15.3 to 45.8)	0.00	0.0%
Test for subgroup differences	Q = 26.95, df = 3		*P* = 0.0026	
**Symptoms**				
No data on symptoms reported	k = 8 (47)	12.8% (5.85 to 25.6)	0.00	0.0%
Pre or asymptomatic	k = 11 (85)	31.8% (22.8 to 42.4)	17.21	41.9%
Symptomatic	k = 31 (249)	46.2% (40.1 to 52.4)	19.79	0.0%
Test for subgroup differences	Q = 18.34, df = 2		*P* < 0.001	
**Vaccination status**				
Vaccination status not reported	K = 16 (118)	44.1% (35.4 to 53.1)	9.95	0.0%
No vaccination	K = 21 (221)	33.0% (27.1 to 39.5)	24.73	19.1%
Yes	K = 4 (42)	54.8% (39.7 to 69.0)	3.6	16.6%
Test for subgroup differences	Q = 8.80, df = 2		*P* = 0.012	
**Platform**				
N gene	k = 15 (168)	35.1% (28.3 to 42.6)	25.3	44.7%
E gene	k = 14 (80)	46.3% (35.7 to 57.2)	0.41	0.0%
S gene	k = 2 (45)	17.8% (9.15 to 31.7)	2.17	0.0%
Other	K = 10 (88)	50.0% (39.7 to 60.3)	0.02	0.0%
Test for subgroup differences	Q = 14.89, df = 3		*P* = 0.002	
**Hospitalized**				
No data on hospitalization status	k = 4 (78)	43.6% (33.1 to 54.7)	0.51	0.0%
Non-hospitalized	k = 15 (165)	37.0% (30.0 to 44.6)	13.8	0.0%
Yes	k = 29 (138)	38.4% (30.7 to 46.8)	15.6	0.0%
Test for subgroup differences	Q = 0.992, df = 2		*P* = 0.609	
**Intensive care**				
No data on intensive care status	K = 11 (135)	44.4% (36.3 to 52.9)	16.11	44.1%
Non-intensive care	K = 28 (224)	35.7% (29.7 to 42.2)	16.26	0.0%
Yes	K = 9 (22)	36.4% (19.3 to 57.7)	0.40	0.0%
Test for subgroup differences	Q = 2.75, df = 2		*P* = 0.252	
**Medical conditions**				
No data on medical conditions	K = 8 (199)	35.7% (29.3 to 42.6)	15.33	54.3%
Data on medical conditions extracted from papers	K = 35 (182)	42.0% (35.3 to 49.6)	30.18	0.0%
Test for subgroup differences	Q = 1.76, df = 1		*P* = 0.185	
Cancer (including solid and blood)*				
No	K = 20 (135)	31.1% (23.9 to 39.4)	18.15	0.0%
Yes	K = 24 (47)	74.5% (59.5 to 84.5)	4.40	0.0%
Test for subgroup differences	Q = 24.9, df = 2		*P* < 0.0001	
**At least one chronic condition (including diabetes, hypertension, IHD, etc)**				
No	K = 6 (24)	45.8 (27.5 to 65.4)	2.09	0.0%
Yes	K = 32 (158)	41.8 (34.3 to 49.6)	28.54	0.0%
Test for subgroup differences	Q = 1.90, df = 2		*P* = 0.387	
**Immunocompromised (including on immune-suppressive drugs)**				
No	K = 23 (133)	38.3 (30.5 to 46.9)	19.7	0.0%
Yes	K = 19 (49)	53.1% (39.2 to 66.5)	10.6	0.0%
Test for subgroup differences	Q = 4.91, df = 2		*P* = 0.086	
**Transplant**				
No	K = 29 (156)	39.1% (31.8 to 47.0)	21.93	0.0%
Yes	K = 11 (26)	61.5% (42.1 to 77.9)	2.64	0.0%
Test for subgroup differences	Q = 6.10, df = 2		*P* = 0.0470	
**COVID–19 Treatments (Remdesivir, Dexamethasone, etc)**				
No	K = 22 (158)	38.6 (31.3 to 46.4)	28.79	30.5%
Yes	K = 16 (24)	66.7% (46.1 to 82.4)	0.24	0.0%
Test for subgroup differences	Q = 7.86, df = 2		*P* = 0.02	
**Lineage**				
No data on lineage	K = 20 (196)	30. 6 (24.6 to 37.4)	28.07	32.3%
A	k = 5 (7)	57.14 (23.0 to 85.6)	0.00	0.0%
B	k = 15 (103)	59.2 (49.5 to 68.3)	4.52	0.0%
C/Delta	k = 5 (45)	22.2 (12.4 to 36.6)	0.41	0.0%
Omicron	k = 1 (30)	43.3 (27.1 to 61.2)	0.00	-
Test for subgroup differences	Q = 28.67, df = 4		*P* < 0.0001

**Table 2.** Characteristics of included studies Table 2. long description.

Study	Country	Setting	Population/environment	Study individuals’ numbers	Funding
Alshukairi 2021	Saudi Arabia	Hospitalized cases	7 immunocompromised patients and 6 immunocompetent patients	13	None
Avanzato 2021	USA	Long-term care facility	71 years old female, with a 10-year history of chronic lymphocytic leukaemia (CLL), acquired hypogammaglobulinemia, anaemia, and chronic leucocytosis.	1	This work was supported by the Intramural Research Programme of the National Institute of Allergy and Infectious Diseases (NIAID).
Aydillo 2020	USA	Tertiary care cancer centre; HCT and cellular therapy recipients or HCT candidates who were diagnosed with COVID-19 during the peak of the New York City epidemic (10 March – 20 April, 2020)	Immunocompromised patients	11	Supported by an award (P01 CA23766) and a NIH–National Cancer Institute Cancer Center Support Grant (P30 CA008748), a grant (to Drs. Hohl, Babady, and Kamboj) from the Jack and Dorothy Byrne Foundation, a contract from the National Institute of Allergy and Infectious Diseases (HHSN272201400008C) awarded to the Center for Research on Influenza Pathogenesis –(a Center of Excellence for Influenza Research and Surveillance), philanthropic donations from the JPB Foundation, a research grant (2020–215,611 [5384]) from the Open Philanthropy Project, philanthropic donations (to Dr. García-Sastre) from Mount Sinai Philanthropy, awards (S10OD018522 and S10OD026880) from the NIH Office of Research Infrastructure Programmes, and a Robin Chemers Neustein Postdoctoral Fellowship Award.(to Dr. Gonzalez-Reiche
Baang 2021	USA	The patient had 3 admissions related to COVID-19 over a 4-month period	Patient with lymphoma and associated B-cell immunodeficiency	1	This work was supported by a coronavirus disease 2019 (COVID-19) Response Innovation Grant from the University of Michigan. Additional support for the enrollment of hospitalized patients with COVID-19 was provided by the Centers for Disease Control and Prevention (U01 IP000974).
Basheer 2021	Israel	The patient was hospitalized during D25-D41, D61-D70	Immunocompromised patient (due to Rituximab treatment)	1	No external funding
Decker et al. [15]	Germany	Nosocomial COVID-19.	Patient with recent heart transplantation.	1	A. Lother is funded by the Berta-Ottenstein-Programme for Advanced Clinician Scientists, Faculty of Medicine, University of Freiburg.
Gniazdowski 2021	USA	Samples processed at the Johns Hopkins molecular virology laboratory from ICU, hospitalized and outpatients	Repeated testing was identified by pulling the data of all molecular COVID-19 testing that was conducted in the Johns Hopkins Hospital Microbiology laboratory from 11 March to 11 May 2020.	40	This work was supported by the Department of Pathology, Johns Hopkins School of Medicine, the National Institutes of Health (NIH), Johns Hopkins Center of Excellence in Influenza Research and Surveillance (grant HHSN272201400007C to A. P. and H. H. M.), and the Molecular and Cellular Basis of Infectious Diseases programme, NIH (grant T32A1007417 to H. P. and B. S.).
Guetl et al. [16]	Austria	Hospitalized D16-D55	Patient with X chromosome-linked agammaglobulinemia (XLA), obstructive respiratory disorder, impaired alveolar diffusion capacity and non-cystic fibrosis bronchiectasis.	1	This research did not receive any specific grant from funding agencies in the public, commercial, or not-for-profit sectors
Han 2021	USA	Hospital	Patient with recent allogeneic peripheral blood stem cell transplant (PBSCT) (after 38 days), with CMV and graft failure	1	None
Jung et al. [21]	Korea	Unknown source of infection.	Health care workers (HCWs)	32	This work was supported by the Korea Health Technology R&D Project through the Korea Health Industry Development Institute (KHIDI) funded by the Ministry of Health & Welfare, South Korea (grant number HD22C2045); and the National Research Foundation of Korea (NRF) funded by the Ministry of Science and ICT, Republic of Korea (grant number NRF-2022M3A9I2017241).
Ke 2021	USA	Unknown source of infection.	Faculty, staff, and students	60	This work was supported by the National Heart, Lung, and Blood Institute at the National Institutes of Health [3U54HL143541-02S2] through the RADx-Tech programme. The views expressed in this manuscript are those of the authors and do not necessarily represent the views of the National Institute of Biomedical Imaging and Bioengineering; the National Heart, Lung, and Blood Institute; the National Institutes of Health, or the U.S. Department of Health and Human Services.
Kim 2022a	Korea	Tertiary hospital	Patients with confirmed COVID-19 who agreed to multiple serial sampling	20	Grants from the Korea Health Technology R&D Project through the Korea Health Industry Development Institute (KHIDI), which is funded by the Ministry of Health & Welfare, Republic of Korea (grant No. HW20C2062) and from the National Research Foundation of Korea (NRF) funded by the Ministry of Science and ICT, Republic of Korea (NRF-2018M3A9H4056537).
Kujavski 2020	USA	Non-hospitalized and hospitalized patients	Non-hospitalized cases (n = 2; P1 and P5); hospitalized cases (n = 7; P6-P12)	9	N/R
Laferl 2020	Austria	HCWs in self isolation because of a PCR-confirmed SARS-CoV-2 infection	Healthy HCWs off work because of ongoing positive RT-PCR results in combined NP and OP swabs following SARS-CoV-2 infection	15	None
Lang 2020	Austria	Community exposure	Patient with severe COVID-19	1	N/R
Leitão 2021	Brazil	N/R	Mildly symptomatic immunocompetent cases with long-lasting positive RT-PCR	51	This work was supported by Conselho Nacional de Desenvolvimento Científico e Tecnológico, Fundação Carlos Chagas Filho de Amparo à Pesquisa do Estado do Rio de Janeiro, Coordenação de Aperfeiçoamento de Pessoal de Nível Superior, Instituto Serrapilheira, Ministério da Ciência, Tecnologia e Inovações, Coordenação de Projetos, Pesquisas e Estudos Tecnológicos, and Universidade Federal do Rio de Janeiro.
Leung 2022	Canada	Community exposure	Relapsed, refractory B-cell acute lymphocytic leukaemia (ALL)	1	None.
Lin et al. [5]	Canada	Hospital, community	Hospitalized and community patients	27	This publication was funded in part through Alberta Health Services, Alberta Precision Laboratories Internal Operating Funds, the University of Calgary Infectious Diseases Research and Innovation Fund for COVID-19, the Canadian Institutes for Health Research, and the University of Alberta’s Li Ka Shing Institute of Virology.
Lu 2020	China	Quarantine facility - the patients lived in a well-ventilated single room, dined separately, practiced hand hygiene, and minimized close contact with others.	Discharged COVID-19 cases were isolated in designated hotels for another 14 days.	16	This work was supported by grants from Guangdong Provincial Novel Coronavirus Scientific and Technological Project (2020111107001), China Evergrande Group (2020GIRHHMS11), Science and Technology Planning Project of Guangdong(2018B020207006), National Science and Technology Project (2020YFC0846800).
Mancon 2022	Italy	Hospitalized patient.	Patient with follicular lymphoma	1	N/R
Mendes-Correa 2021	Brazil	Patient from community setting. Hospitalized.	Patient with a prior autologous hematopoietic stem cell transplant due to a diffuse large B-cell lymphoma.	1	São Paulo Research Foundation (FAPESP) (Number:2020/05623–0). This work was also supported by a Medical Research Council-São Paulo Research Foundation (FAPESP) CADDE partnership award (MR/S0195/1 and FAPESP 18/14389–0) (caddecentre.org/). N.R. F is supported by Wellcome Trust and Royal Society (Sir Henry Dale. Fellowship: 204311/Z/16/Z).
Mileto 2021	Italy	N/R	Patient with prostatic cancer	1	None
Monrad et al. [11]	Denmark	Patient from community setting. Hospitalized.	Patient with chronic lymphocytic leukaemia.	1	This work was funded by a research grant from RegionMidt/Aarhus University Hospital and Aarhus University.
Murata 2021	Japan	Cruise ship	Passengers from cruise ship; asymptomatic SARS-CoV-2 carriers who had two or more positive RT-PCR test results at the hospital were analysed for the presence of viable virus using cell culture	39	Japan Agency for Medical Research and Development (AMED) under grants JP19fk0108150 and JP20fk0108150.
Nissen 2021	Sweden	Nursing home	Healthy HCW exposed to SARS-CoV-2 infection	1	Swedish Research Council (VR: 2017–05807, 2018–02569); The European Union’s Horizon 2020 Research Innovation Program, (grant 874,735 (VEO)); The Knut and Alice Wallenberg Foundation; and the Science for Life Laboratory Uppsala (Projects: Nevermore Covid and SiCoV).
Niyonkuru 2021	Denmark	Patient 1 - community. Patient 2 - hospital acquired	Immunocompromised patients	2	This work was supported by a Novo Nordisk grant, number [NNF20SA0062931].
Nomura et al. [12]	Japan	Patients admitted for SARS-CoV–2 infection	Severe COVID-19 cases	6	The authors received no funding for this study.
Pedro 2021	Portugal	Patient admitted for SARS-CoV-2 infection	Patient from community; contact with COVID-19 cases	1	Portuguese Foundation for Science and Technology. i3S is supported by FEDER—Fundo Europeu de Desenvolvimento Regional funds through the COMPETE 2020—Operational Program for Competitiveness and Internationalization (POCI), Portugal 2020, and by Portuguese funds through FCT/Ministério da Ciência, Tecnologiae Inovação in the framework of the project ‘Institute for Research and Innovation in Health Sciences’ (POCI-01-0145-FEDER-007274).
Pérez-Lago 2021	Spain	Hospitalized cases. Probably community acquired	Cases admitted for COVID-19, with immunosuppression	3	This research was funded by Instituto de Salud Carlos III (Ref COV20/00140: SeqCOVID— Consorcio para la epidemiología genómica de SARS-CoV-2 en España) and by Consejo Superior de Investigaciones Científicas (CSIC) (PTI Salud Global). Miguel Servet Contracts CP15/00075 and CPII20/00001) to L.P.-L.
Pickering 2021	UK	Hospitalized cases	Inpatients, admitted for COVID-19 (one case) and other conditions (4 cases)	5	King’s Together Rapid COVID-19, Medical Research Council, Wellcome Trust, Huo Family Foundation, UK Department of Health, National Institute for Health Research Comprehensive Biomedical Research Centre.
Rajakumar 2021	Canada	Case 1: Community exposure. Case 2: unknown source of infection	Recent cardiac transplant patients	2	This publication was funded in part by a grant from Alberta Health Services.
Sepulcri et al. [35]	Italy	N/R	Recently diagnosed with non-Hodgkin lymphoma (mantle cell type, blastoid variant stage IV). Completed 2 cycles of rituximab, bendamustine, cytarabine. Lymphoma in remission	1	The authors received no specific funding for this work.
Siedner et al. [18]	USA	Ambulatory individuals with postvaccination breakthrough infections. HCW, community	Non-hospitalized individuals with confirmed SARS-CoV-2 infection after vaccination. Employees in the Mass General Brigham, all individuals with positive SARS-CoV-2 PCR test results in the Mass General Brigham Medical System.	22	N/R
Singh et al. [19]	USA	Patients from a skilled nursing facility or rehabilitation facility	Hospitalized congregate care patients being treated at Albany Medical Centre	14	N/R
Sung 2022	USA	Tertiary referral hospital.	Immunocompromised adults	12	his study was supported by the CDC Prevention Epi-Center Grant: 5U54CK000482. JK is supported by the grant 1K23AI137321-01A1 from the National Institute 320 of Allergy and Infectious Diseases. MD is supported by R01 AI157155.
Tarhini 2021	France	Patients from community. Patient 3 - homeless	Immunocompromised Patients	3	This study was funded in part by the Agence Nationale de la Recherche sur le SIDA et les Hépatites Virales (ANRS), the PhyloCoV study, funded by the Fondation pour la Recherche Médicale (FRM), and the TheraCoV study, funded by the Agence Nationale pour la Recherche (ANR).
Thornton 2022	Canada	Hospital. Patient from community.	Patient with follicular lymphoma 10 months prior and received 6 cycles of chemo-immunotherapy (bendamustine and rituximab), achieving clinical remission; he received an additional dose of maintenance rituximab one month prior.	1	Not applicable
Truong 2021	USA	Unknown source of infection. Hospitalized.	Immunosuppressed patients with B-cell acute lymphoblastic leukaemia.	3	The funders had no role in study design, data collection, data analysis, data interpretation, or writing of the report.
Weigang 2021	Germany	Unknown source of infection. Hospitalized.	Immunosuppressed patient with the first positive RT-qPCR positive result 12 days after kidney transplantation.	1	Open Access funding enabled and organized by Projekt DEAL.
Williamson 2021	UK	Unknown source of infection. Hospitalized.	Immunocompromised patient.	1	Funded by the Southmead Hospital Charity. Immunological work was funded by an Elizabeth Blackwell Institute TRACK award. A.D.D. / D.A.M are supported by the United States Food and Drug Administration (HHSF223201510104C) and the UK Research and Innovation / Medical Research Council (MRC) and Biotechnology and Biological Sciences Research Council (grants MR/V027506/1 and BB/V013874/1). M.K.W is supported by MRC grant MR/V027506/1 (awarded to A.D.D). COG-UK is supported by funding from the MRC part of UKRI, the National Institute of Health Research (NIHR) and Genome Research Limited, operating as the Wellcome Sanger Institute. The authors would like to acknowledge support of the University of Bristol’s Alumni and Friends, which funded the ImageXpress Pico Imaging System. A.D.D and D.A.M. are members of the G2P-UK National Virology consortium funded by MRC/UKRI (grant MR/W005611/1.)
Zahn 2021	Germany	A physician-led medical service (Internal Medical Service, IMS) was established at the Paul Ehrlich Institute (PEI) in Langen/Hessen/ Germany to conduct routine and occasion-based testing of employees	HCW	3	N/R
Zupin 2022	Italy	N/R	HCW	1	This work was supported by IRCCS Burlo Garofolo/Italian Ministry of Health (RC 15/2017, 03/2020, 47/2020).
Garcia-Knight [13]	USA	Index case and household members	Outpatients recruited if they were within 5 days of symptom onset, had at least 1 household member (HM) and had no HMs with COVID-19 symptoms in the preceding week. Participants under 18 years of age and adults who were immunocompromised, hospitalized or who had received monoclonal antibody therapy, were excluded.	82	The FindCOVID study at UCSF is funded by the Centers for Disease Control and Prevention (CDC Contract FE_08009). JDK was supported during this study by the National Institute of Allergy and Infectious Diseases (K23 grant number AI146268). The funders had no role in study design, data collection and analysis, decision to publish, or preparation of the manuscript.
Kang et al. [20]	Korea	Healthcare workers (HCWs) with positive SARS-CoV-2 infection test results. Outpatients	Healthcare workers (HCWs) with positive SARS-CoV-2 infection test results	82	Grants from the research fund donation for COVID-19 research to Asan Medical Center by Kyu-Kang Cho, the Korea Health Technology R&D Project through the Korea Health Industry Development Institute (KHIDI), which is funded by the National Institute of Infectious Diseases, National Institute of Health, Republic of Korea (grant No. HD22C2045), and the National Research Foundation of Korea (NRF) funded by the Ministry of Science and ICT, Republic of Korea (NRF 2022M3A9I2017241).
Kim 2022b	USA	Unknown source of infection. Patients admitted to hospital	Severe or critical COVID-19	5	This work was supported in part by a COVID-19 supplemental grant to Centers for Disease Control and Prevention (CDC) cooperative agreement U54 CK000481-05-02. D.K. is a recipient of Leadership in Epidemiology, Antimicrobial stewardship, Public health (LEAP) fellowship training award sponsored by SHEA, IDSA, PIDS and received training grant from IDSA.
Luna-Muschi 2022	Brazil	HCWs with mild COVID-19 diagnosed by RT-PCR or RAT within 5 days of symptom onset	HCWs	30	This study was supported by the Itaú Unibanco “Todos pela saúde” programme
McCormick 2023	USA	Outbreak in a congregate setting	Incarcerated persons	11	This project was funded by the Centers for Disease Control and Prevention
Tobolowski 2022	USA	149-bed nursing home	Nursing home residents undergoing CDC monitoring study (PPS) that were periodically tested for SARS-CoV-2 resulting positive for COVID-19	11	The work did not receive any non-CDC funding support
Spinicci 2022	Italy	Home, household contact	57-year-old Italian man with 9-month history of eosinophilic granulomatosis with polyangiitis (EGPA)	1	Open access funding provided by Università degli Studi di Firenze within the CRUI-CARE Agreement. This work was supported by funds from the Ministry of Education, University and Research (Italy) Excellence Departments 2018–2022 (Project for the Department of Experimental and Clinical Medicine).
Choi et al. [14]	USA	Unknown source of infection.	Patient with a history of catastrophic antiphospholipid syndrome (APS), complicated by venous thromboembolic, pulmonary emboli, thrombotic microangiopathy, adrenal haemorrhage, coronary vasculitis, and aortitis.	1	N/R

**Table 3.** Quality of included studies Table 3. long description.

Study	Were the criteria for diagnosing a case clearly reported and appropriate	Was the reporting of patient/population characteristics adequate	Were the methods used to obtain RT-PCR results replicable, generalizable and appropriate?	Were the methods used to obtain viral culture results replicable and appropriate	Was the study period, including follow-up, sufficient	Notes
Alshukairi 2021	Unclear	No	Unclear	Unclear	Yes	Case definition unclear; the article reports positive RT-PCR, but Ct cutoff not reported. Data on clinical symptoms lacking. No data on RT-PCR controls. The cell line used was not one that is demonstrated to support SARS-CoV-2 growth. Viral cultures - no data on exclusion of contamination or co-infection (use appropriate antibacterials and antimycotics); no information on confirmatory testing with immunostaining or supernatant PCR testing.
Avanzato 2021	Yes	Yes	Unclear	Yes	Yes	No data on RT-PCR controls. Ct up to 40
Aydillo 2020	Yes	Yes	Unclear	Yes	Yes	Ct up to 40. The information on RT-PCR is limited; the authors used a modified Centers for Disease Control and Prevention (CDC) protocol. No data on RT-PCR controls.
Baang 2021	Unclear	Yes	Unclear	Unclear	Yes	Case definition unclear; the article reports positive RT-PCR, but Ct cutoff not reported. No data on RT-PCR controls. Viral cultures - no data on preparation, or media used; no data on exclusion of contamination or co-infection (use appropriate antibacterials and antimycotics).
Basheer 2021	Yes	Unclear	Yes	Yes	Yes	Data on clinical symptoms are sparse.
Choi et al. [14]	Yes	Yes	Yes	Yes	Yes	Ct value <40.
Decker et al. [15]	Unclear	Yes	Unclear	Unclear	Yes	Case definition unclear; the article reports positive RT-PCR, but Ct cutoff not reported. No information on the PCR test type / platform used. No information on RT-PCR gene or controls. Limited information on viral cultures methods.
Garcia-Knight et al. [13]	Yes	No	Yes	Yes	Yes	Ct cutoff of 40. Data on clinical symptoms are sparse.
Gniazdowski 2021	Unclear	No	Unclear	Yes	Yes	Case definition unclear. Data on clinical symptoms are sparse. The authors used 2 molecular diagnostic assays for SARS-CoV-2, but they do not provide the specific data, explaining that their data indicate that Ct values are comparable for the 2 genes.
Guetl et al. [16]	Unclear	Yes	Unclear	Unclear	Yes	No data on PCR test type or platform used. No data on RT-PCR type, gene used, or controls. No data on culture methods. Case report.
Han 2021	Unclear	Yes	Unclear	Unclear	Yes	Case definition unclear; the article reports positive RT-PCR, but Ct cutoff not reported. No data on RT-PCR controls. No data on culture methods
Jung et al. [21]	Yes	Unclear	Yes	Unclear	Yes	Data on clinical symptoms are sparse. Viral cultures - no data on preparation, or media used; no data on exclusion of contamination or co-infection (use appropriate antibacterials and antimycotics).
Kang et al. [20]	Yes	No	Yes	Unclear	Yes	Ct cutoff of 40. No information on chronic conditions or degree of immunosuppression. No information on exclusion of contamination or co-infection (use appropriate antibacterials and antimycotics). No information on viral cultures controls.
Ke 2021	Yes	Yes	Unclear	Unclear	Yes	No information on the RT-PCR gene. No information on culture controls.
Kim 2022a	Yes	No	Yes	Unclear	Yes	Data on clinical symptoms are sparse. Viral cultures - no information on exclusion of contamination or co-infection (use appropriate antibacterials and antimycotics)
Kim 2022b	Yes	Yes	Yes	Yes	Yes	Ct <40 (reference 17)
Kujavski 2020	Unclear	Yes	Unclear	Unclear	Yes	Case definition unclear; the article reports positive RT-PCR, but Ct cutoff not reported. No information on the PCR test type or platform used. No information on viral cultures controls.
Laferl 2020	No	No	Yes	Unclear	Yes	A SARS-CoV-2 Ct-value of >42 was considered a negative result. Data on clinical symptoms are sparse. Viral cultures - no information on exclusion of contamination or co-infection (use appropriate antibacterials and antimycotics). Information on confirmatory testing with immunostaining or supernatant PCR testing not reported
Lang 2020	Unclear	Yes	Unclear	Unclear	Yes	Case definition unclear; the article reports positive and negative RT-PCR, but Ct cutoff not reported. No information on the PCR test type / platform used. No information on RT-PCR gene or controls. Limited information on viral cultures methods.
Leitão 2021	Yes	No	Unclear	Yes	Yes	Data on clinical symptoms are sparse. No information on PCR controls.
Leung 2022	Unclear	Yes	Unclear	Unclear	Yes	Case definition unclear; the article reports positive RT-PCR, but Ct cutoff not reported. No information on the PCR test type / platform used. No information on RT-PCR controls. The information on viral cultures methods is limited.
Lin et al. [5]	Yes	Yes	Yes	Yes	Yes	
Lu 2020	Unclear	No	Unclear	Unclear	Yes	Case definition unclear; the article reports positive or negative RT-PCR, but Ct cutoff not reported. The data on symptoms is limited. No information on RT-PCR controls. Limited information on viral cultures.
Luna-Muschi 2022	Yes	No	Unclear	Unclear	Yes	Ct value ≤35. The first day of symptoms was considered day 1. No information on the RT-PCR controls. The degree of immunosuppression not reported. No information on viral cultures controls.
Mancon 2022	Unclear	Yes	Unclear	Unclear	Yes	Case definition unclear; the article reports positive or negative RT-PCR, but Ct cutoff not reported. No information on RT-PCR controls. Limited information on viral cultures
McCormick 2023	Unclear	No	Unclear	Unclear	Yes	Case definition unclear; the article reports positive and negative RT-PCR, but Ct cutoff not reported. No information on RT-PCR gene or controls. Data on symptoms not available in all subjects. No information on exclusion of contamination or co-infection (use appropriate antibacterials and antimycotics), and controls.
Mendes-Correa 2021	Unclear	Yes	Unclear	Unclear	Yes	Case definition unclear; the article reports positive or negative RT-PCR, but Ct cutoff not reported. No information on RT-PCR controls. Viral cultures - no information on exclusion of contamination or co-infection (use appropriate antibacterials and antimycotics).
Mileto 2021	Yes	Yes	Unclear	Unclear	Yes	Ct value <43 (limit of detection). No information on RT-PCR controls. Limited information on viral cultures methods.
Monrad et al. [11]	Unclear	Yes	Unclear	Yes	Yes	Case definition unclear; the article reports positive RT-PCR, but Ct cutoff not reported. No information on the PCR test type / platform used.
Murata 2021	Yes	Yes*	Unclear	Yes	Yes	Cp cutoff = 40. Only typical symptoms were assessed. Olfactory and gustatory impairment was not recognized as characteristic of COVID-19 at the time and was not routinely checked (Asymptomatic cases). No information on the PCR test type / platform used, RT-PCR gene, and RT-PCR controls.
Nissen 2021	Unclear	Unclear	Unclear	Yes	Yes	Case definition unclear; the article reports positive and negative RT-PCR, but Ct cutoff not reported. Veracity of symptom & sign checking not reported (pre-symptomatic case). No information on the RT-PCR controls.
Niyonkuru 2021	Unclear	Unclear	Yes	Unclear	Yes	Case definition unclear; the article reports positive RT-PCR, but Ct cutoff not reported. Veracity of symptom & sign checking not reported (pre-symptomatic and asymptomatic case). No information on exclusion of contamination or co-infection (use appropriate antibacterials and antimycotics).
Nomura et al. [12]	Unclear	No	Yes	Unclear	Yes	Case definition unclear; the article reports positive RT-PCR, but Ct cutoff not reported. Limited information on clinical symptoms/signs. Limited information on viral cultures methods.
Pedro 2021	Unclear	Yes	Yes	Unclear	Yes	Case definition unclear; the article reports positive RT-PCR, but Ct cutoff not reported. Limited information on viral cultures methods.
Pérez-Lago 2021	Unclear	Yes	Unclear	Yes	Yes	Case definition unclear; the article reports positive RT-PCR, but Ct cutoff not reported. No information on the RT-PCR gene, and RT-PCR controls.
Pickering 2021	Yes	No	Unclear	Yes	Yes	Ct cutoff 40. Veracity of symptom & sign checking not reported (asymptomatic cases). No information on the RT-PCR controls.
Rajakumar 2021	Yes	Yes	Yes	Yes	Yes	
Sepulcri et al. [35]	Yes	Yes	Unclear	Unclear	Yes	Ct value for positive RT-PCR: 41 cycles. No information on the RT-PCR controls. Limited data on the viral culture methods
Siedner et al. [18]	Unclear	No	Yes	Yes	Yes	Case definition unclear; the article reports positive RT-PCR, but Ct cutoff not reported. Symptomatic infections were defined as those with COVID-19–related symptoms at any point during the observation period. No information if the patient was pre-symptomatic.
Singh et al. [19]	Unclear	Yes	Unclear	Yes	Yes	Case definition unclear; the article reports positive and negative RT-PCR, but Ct cutoff not reported. No information on the RT-PCR controls.
Spinicci 2022	Yes	No	Unclear	Unclear	Yes	Ct value ≤40. Data on clinical symptoms are sparse. No information on RT-PCR gene or controls. No information on exclusion of contamination or co-infection (use appropriate antibacterials and antimycotics), and controls.
Sung 2022	Yes	No	Unclear	Yes	Yes	Ct up to 45. No information on specific RT-PCR test type. No information on the RT-PCR controls. Limited information on symptoms.
Tarhini 2021	Yes	Yes	Unclear	Unclear	Yes	Ct up to 40. No information on the RT-PCR controls. No information on exclusion of contamination or co-infection (use appropriate antibacterials and antimycotics).
Thornton 2022	Yes	Yes	Unclear	Unclear	Yes	Ct cutoff of 40. No information on the RT-PCR controls. No information on viral cultures controls.
Tobolowski 2022	Yes	No	Unclear	Unclear	Yes	Ct value ≤40. Limited information on symptoms / signs. No information on RT-PCR controls. No information on viral culture controls
Truong 2021	Unclear	Unclear	Unclear	Unclear	Yes	Case definition unclear; the article reports positive and negative RT-PCR, but Ct cutoff not reported. Veracity of symptom & sign checking not reported (pre-symptomatic cases). No information on RT-PCR gene, and RT-PCR controls. No information on exclusion of contamination or co-infection (use appropriate antibacterials and antimycotics), and controls.
Weigang 2021	Yes	Yes	Yes	Yes	Yes	
Williamson 2021	Unclear	Unclear	Unclear	Yes	Yes	Case definition unclear; the article reports positive and negative RT-PCR, but Ct cutoff not reported. Limited information on symptoms / signs. No information on RT-PCR controls.
Zahn 2021	Yes	No	Yes	Yes	Yes	Ct detection limit 40. Veracity of symptom & sign checking not reported (asymptomatic case).
Zupin 2022	Unclear	No	Yes	Yes	Yes	Case definition unclear; the article reports positive and negative RT-PCR, but Ct cutoff not reported. Veracity of symptom & sign checking not reported (pre-symptomatic case)

**Table 4.** Characteristics of the analysis set based on the first RT-PCR test result (the first test result is used except for the Number of observations and the length of time following the first RT-PCR*) Table 4. long description.

Characteristic	Value
Number of patients	N = 381
Number of studies	N = 39
Number of observations (per patient)*	Median 3 IQR (2–7)
Length of time following first RT-PCR *	Median 5 IQR (0–11)
Number (%) of patients who were culture-positive test at first test	148 (38.8%)
Number (%) of patients who were symptomatic at first test	253 (66.4%)

**Table 5.** Multivariable model. Independent associations with cycle threshold values Table 5. long description.

Characteristic	Change in Ct (95% CI)	*P* value
Overall average at reference levels	30.8 (27.8, 33.8)	
***Culture***		
Negative	Ref level	*P* < 0.001
Positive	−6.55 (−7.83 to −5.26)	
***Time since the first PCR***		
< 7 days	Ref level	*P* < 0.001
7–13 days	+2.37 (0.622 to 4.080)	
14–28 days	+4.06 (2.04 to 6.06)	
28 days +	+5.96 (3.18 to 8.69)	
***COVID 19 treatments***		
No data on treatments	Ref level	*P* < 0.001
No mention	0.45 (−2.07 to 2.98)	
Yes	−5.51 (−8.15, −2.00)

**Table 6.** Multivariable model. Independent associations with culture positivity Table 6. long description.

Characteristic	Odds ratio (approx 95% CI)	*P* value
CT value	0.84 (0.804 to 0.877)	*P* < 0.0001
Time since the first PCR		
< 7 days	Ref level	*P* = 0.005
7–13 days	0.54 (0.267 to 1.11)	
14–28 days	0.286 (0.139 to 0.589)	
28 days +	0.635 (0.184 to 2.19)	
***Cancer***		
No clinical data reported	Ref level	*P* = 0.0002
No cancer	1.23 (0.691 to 2.19)	
Cancer	5.59 (2.34 to 13.3)	

## Discussion

We reviewed the largest cohort of studies reporting viral culture and serial RT-PCR testing: 50 studies, with 39 incorporated in quantitative synthesis. We found a significant decrease in culture-positivity percentage when the Ct value increases, correlated with time since the first RT-PCR test (*P* < 0.01).

The percentage of culture-positive samples was higher in the first week after the first RT-PCR, decreasing thereafter (Q = 26.95, *P* = 0.0026). Research on Ct dynamics of other acute respiratory viruses reported Ct values of 25–30 on the day of symptom onset, lower over the ensuing 1–3 days, and progressively higher up to ≥30 after 1 week for most viruses, mirroring patterns seen of SARS-CoV-2 infection [25]. Rhinovirus Ct values were higher and more stable over time [25]. These findings may be attributed to challenges of identifying the first day of symptoms in mild infections (e.g., rhinovirus), in contrast to more severe diseases such as influenza with abrupt onset. Symptoms of respiratory virus infections, including SARS-CoV-2, are well-described and can naturally fluctuate over time [26, 27].

The high rate of culture positivity at first testing (38.8%) could be explained by early testing post symptom onset and/or post exposure. Human challenge studies on SARS-CoV-2 in healthy subjects reported a steep rise in culturable virus in the nose and throat after inoculation, peaking at approximately 5 days [28]. After a brief plateau, the culturable virus declined rapidly, although very low levels persisted in some subjects to day 12 in the nose. Quantifiable RNA by qPCR lingered longer and was still present on day 14 after inoculation. At these later time points low-level qPCR positivity remained in 33% of participants at day 28 after inoculation [28]. Between 2020 and 2023, different SARS-CoV-2 strains circulated, some of which, like Omicron, were more difficult to culture [29]. Some bias might exist in included studies, as methods for viral cultures were unclear in 22/39 (56.4%) studies, and methods of RT-PCR testing were unclear in 29/39 (74.4%) studies.

The presence of COVID-19 symptoms was associated with an increased proportion of culture-positivity (Q = 18.34, *P* < 0.01), but verification of symptoms was not reported in 22/39 (56.4%) studies. Symptom ascertainment bias was higher earlier in the pandemic [8], especially in the very elderly and those with cognitive impairment [27, 30].

Patients with different cancer types, including solid and blood malignancies, had a higher proportion of positive viral cultures (Q = 24.9, *P* < 0.0001), consistent with previous findings [31, 32]. We combined all types of cancers due to a limited number of reports and our definition of immunocompromised was deliberately broad to avoid bias towards any subgroup [33, 34]. The immunocompromised subgroup had a longer infection duration, with no significant difference in the non-B-cell malignancy group (0.58 [95% CI 0.31–1.09]) [32]. The most prolonged interval of positive viral cultures was on day 238 in a patient with non-Hodgkin lymphoma (mantle cell type, blastoid variant stage IV) [35]. We found no subgroup interaction for hospitalization or admission in intensive care units. Various criteria worldwide restrict our confidence in the absence of an association with hospital admissions, especially early in the pandemic, when the admission threshold was lower.

Shah, 2021 found no significant difference in mean Ct values between hospitalized and non-hospitalized patients in line with studies for other pathogens [36–38].

We found no subgroup interaction for the presence of any chronic conditions such as diabetes, hypertension, or IHD, but COVID-19 treatments (e.g., remdesivir, dexamethasone) significantly reduced Ct (adjusted change in Ct −6.02, 95%CI, −9.65 to −2.48, *P* = 0.002). No firm conclusions could be drawn on vaccination as we only found data on 42/381 patients.

It was not possible to address the influence of age, sex, specific underlying pathologies (e.g., diabetes, hypertension), immunosuppression degree, and laboratory variables on infectiousness, as heterogeneity and limited available individual data precluded performing analyses.

Two-stage meta-analysis showed that association with culture-positivity was higher for the E gene (46.3%; 95% CI 35.7 to 57.2) than for N, S, or other genes used for RT-PCR (Q = 14.89, *P* = 0.002). Our findings suggest that when the Ct value increases by approximately 6.6 units, the culture positivity rate decreases significantly. This relationship was observed across studies using different PCR platforms; however, Ct values may vary between assays, and therefore, such thresholds should be interpreted within the context of the specific testing platform and laboratory methods used. This may be a valuable and reasonable proxy to rule out infectious SARS-CoV-2, as there is a consistent correlation between a rising Ct value and a decreased likelihood of isolating replication-competent virus. Such a value is helpful guiding clinicians in correlation with other clinical and ancillary data.

Our results agree with the findings of previous systematic reviews on infectious potential periods [39–45]. Not all the systematic reviews cross correlated Ct values with viral culture, instead focusing on viral load and did not consistently differentiate immunocompetent from immunocompromised patients. In addition, none of the previous systematic studies provided the detailed rigour of the analytics which we addressed or performed a two-stage meta-analysis. Our analysis only included studies with high-quality evidence, integrating clinical, epidemiologic, molecular, and laboratory data, narrowing uncertainty over the potential SARS-CoV-2 infectiousness and transmission dynamics [46]. Robust research requires thorough serial symptom screening supported by high confirmatory evidence, such as viral culture or longitudinal serial PCRs, to establish replicating and/or infectious virus presence [8, 31, 46, 47].

Collectively, our systematic review results of the use of serial PCR testing and obtaining repeated Ct values ≥30 in immunocompetent patients in conjunction with detailed clinical assessment can be a valuable tool for assisting clinicians in decision-making regarding the risk for forward transmission and de-isolation. Decreasing harms from excessive isolation that may delay discharge, hinder medical treatment [48, 49] such as delays in procedures or appropriate placement (e.g., waiting for a single room, declines for transfer to rehabilitation or skilled nursing facilities), restricted visitation, and compromised medical care (e.g., admission to medical ward instead of a psychiatric unit) [48]. Avoiding unnecessary isolation could contribute to improved contact with patients by healthcare workers (HCWs), less bed blocking, improved psychosocial status, and reductions in deconditioning and mental health deterioration [50, 51] and less environmental pollution [51] from a reduction in gloves, gowns, masks, and eye protection devices.

### Strengths and limitations

Strengths of the present systematic review include adherence to an established protocol, extensive literature searches, double-checked data extraction, and quality assessment. To our knowledge, this is the largest study on the value of serial Cts and viral culture, with high rigour using internal controls and verification of cultures as SARS-CoV-2, with robust data analysis and interpretation involving a significant amount of clinical, ancillary, and epidemiological knowledge. Additional data for some individuals were included after communicating with the authors.

Limitations of our review include a relatively small number of studies using viral culture and serial RT-PCR with complete data on all individuals, substantial heterogeneity in study design and reporting, and the difficulty in combining data owing to the varied methods used for RT-PCR platforms and culture. Some data were extracted from figures in published articles, potentially resulting in less accurate estimations. Despite prioritizing only studies with high levels of evidence to demonstrate the microbiological and clinical aspects of viral respiratory pathogen transmission, the included research showed a moderate risk of bias, potentially impacting the strength of our findings. Other potential limitations include study populations, timings of investigations, use of various respiratory specimens, and investigation quality constraints between different studies [52]. The studies used multiple RT-PCR platforms, several types of culture cell lines, and varying culture conditions. Data on verification methods for RT-PCR and cell cultures (i.e., internal controls) was inconsistent. Several factors affecting SARS-CoV-2 isolation were sometimes underreported (e.g., culturing techniques, transport details from the bedside to the laboratory, and sample storage conditions).

Standardization of SARS-CoV-2 isolation using cell culture and RT-PCR methods is essential for evidence development. Laboratories should consistently use specific platforms and internal standards to calibrate the relation between Ct values and genome copies. A consistent format for case series and longitudinal research is essential to prevent data loss, and observation windows should be limited to 3 days during the acute infection phase. Each observation period should describe symptoms, interventions, and Ct values. With Cts <30, researchers should provide information on viral culture, if available. Patient descriptions should include medical history, interventions, and medication interactions. Investigators should indicate reasons for admission, discharge, and isolation changes. Studies should specify the duration of viral shedding by documenting the time between the first positive and negative viral cultures.

### Implications

Hospital and intensive care unit (ICU) stay is not necessarily a proxy for infectiousness as by the time the individual has arrived in the ICU, they are often post-infectious unless they acquire an infection in the hospital or the ICU or are immunocompromised for any reason. Unless patients are immunocompromised, Ct values rise in line with virus clearance. The findings from this review with serial Ct values using multiple platforms and primer sets, demonstrating a rise over time at threshold cut-offs of ≥30 and the association with negative viral cultures, used in conjunction with clinico-epidemiologic parameters, may assist clinicians and infection prevention and control personnel in informing de-isolation decisions when interpreted alongside clinical context, assay characteristics, and other relevant laboratory data providing objective criteria for infectiousness and risk of forward transmission. However, as research has demonstrated, clearing residual RNA debris may take longer often giving persistently positive high Ct values.

## Conclusion

We found a positive relationship between lower Ct, positive viral culture likelihood, and the symptom onset date. When done serially, preferably using the same platform, a consistent reduction or rise in the Ct value can be used to assess infectiousness status, taking account of several clinical variables that can be utilized to assess infectiousness. This work should feed into guidelines to facilitate interpreting PCR results and could be applied to several other acute respiratory viruses.

## Supporting information

10.1017/S0950268826101484.sm001 Rosca et al. supplementary material Rosca et al. supplementary material

## Data Availability

All data included in this review are derived from previously published studies and are provided within the manuscript tables and supplementary materials.

## Supplementary material

The supplementary material for this article can be found at http://doi.org/10.1017/S0950268826101484.

## Long descriptions

### Figure 1. Long description

At the top, two boxes represent sources: left, ‘Records identified through database searching to 10 September 2020’ with n equals 145; right, ‘Searches from previous reviews on asymptomatic to 04/10/2020, and immunocompromised to 24/03/2022. New searches until 30 June 2025’ with n equals 2,837. Both flow downward to ‘Records after duplicates removed’ (n equals 145 left, n equals 2,837 right). Each proceeds to ‘Records screened’ (n equals 145 left, n equals 2,837 right). Left branch: 116 records excluded, 29 full-text articles assessed for eligibility. Right branch: 2,670 records excluded, 167 full-text articles assessed for eligibility. From the right, 146 records excluded not meeting inclusion criteria. Both branches converge to ‘Studies included in systematic review’ (n equals 50), then to ‘Studies included in quantitative synthesis (meta-analysis)' (n equals 39). Footnotes reference three studies for search details.

Navigate back to Figure 1..

### Figure 2. Long description

From top to bottom, each horizontal bar represents a criterion. The top bar, ‘Was the study period, including follow-up, sufficient,’ is entirely green, indicating all studies answered Yes. The second bar, ‘Were the methods used to obtain viral culture results replicable and appropriate,’ is about one-third green (Yes) and two-thirds yellow (Unclear). The third bar, ‘Were the methods used to obtain R T dash P C R results replicable, generalizable and…,’ is about one-fifth green (Yes) and four-fifths yellow (Unclear). The fourth bar, ‘Was reporting of patient forward slash population characteristics adequate,’ is about half green (Yes), one-sixth yellow (Unclear), and one-third red (No). The bottom bar, ‘Were the criteria for diagnosing a case clearly reported and appropriate,’ is about one-third green (Yes), two-thirds yellow (Unclear), and a small red segment (No) at the end. The x axis is labeled ‘Percent of studies’ from 0 percent to 100 percent. The color legend at the bottom indicates green for Yes, yellow for Unclear, and red for No.

Navigate back to Figure 2..

### Figure 3. Long description

The x-axis shows C T value from 10 to 45. The y-axis shows probability of culture positivity from 0 to 1.0. Five dashed lines are plotted: black circles for first P C R less than 7 days, cancer equals n; red triangles for first P C R 7 to 13.9 days, cancer equals n; green plus for first P C R 14 to 27.9 days, cancer equals n; blue x for first P C R greater than or equal to 28 days, cancer equals n; magenta diamonds for first P C R less than or equal to 7 days, cancer equals y. All lines show a decreasing trend as C T value increases. The magenta line (cancer equals y) remains highest across all C T values, while the green line (first P C R 14 to 27.9 days, cancer equals n) is lowest. The legend is at the top right.

Navigate back to Figure 3..

### Table 1. Long description

Starting from the top, the table is divided into sections by bolded subgroup labels. For CT value, culture positivity decreases as CT increases: less than 20, 74.2 percent; 20 to 24, 64.3 percent; 25 to 29, 24.2 percent; 30 to 34, 7.04 percent; 35 or more, 2.99 percent. For time since first PCR, less than 7 days, 51.9 percent; 7 to 13 days, 34.3 percent; 14 to 27 days, 21.2 percent; 28 or more days, 28.1 percent. For symptoms, no data, 12.8 percent; pre or asymptomatic, 31.8 percent; symptomatic, 46.2 percent. For vaccination status, not reported, 44.1 percent; no vaccination, 33.0 percent; yes, 54.8 percent. For platform, N gene, 35.1 percent; E gene, 46.3 percent; S gene, 17.8 percent; other, 50.0 percent. For hospitalization, no data, 43.6 percent; non-hospitalized, 37.0 percent; yes, 38.4 percent. For intensive care, no data, 44.4 percent; non-intensive care, 35.7 percent; yes, 36.4 percent. For medical conditions, no data, 35.7 percent; data extracted, 42.0 percent. For cancer, no, 31.1 percent; yes, 74.5 percent. For at least one chronic condition, no, 45.8 percent; yes, 41.8 percent. For immunocompromised, no, 38.3 percent; yes, 53.1 percent. For transplant, no, 39.1 percent; yes, 61.5 percent. For COVID-19 treatments, no, 38.6 percent; yes, 66.7 percent. For lineage, no data, 30.6 percent; A, 57.14 percent; B, 59.2 percent; C or Delta, 22.2 percent; Omicron, 43.3 percent. Each section includes the number of studies and patients, 95 percent confidence intervals, Q, and I squared values. Tests for subgroup differences are reported at the end of each section with Q, degrees of freedom, and P values.

Navigate back to Table 1..

### Table 2. Long description

The table contains six columns. The headers from left to right are Study, Country, Setting, Population or environment, Study individuals’ numbers, and Funding. Each row details a study, beginning with the study name, followed by the country, then the setting such as hospital, community, or specific facility. The next column describes the population or environment, specifying patient types or conditions. The fifth column lists the number of individuals included in each study. The final column provides funding information, including grant numbers, supporting institutions, or notes of no funding. For example, the first row lists Alshukairi 2021 from Saudi Arabia, with hospitalized cases, 7 immunocompromised and 6 immunocompetent patients, 13 individuals, and no funding. Other studies span countries such as USA, Israel, Germany, Korea, Brazil, Austria, Italy, Japan, Sweden, Portugal, Spain, UK, China, France, and Canada. Settings include hospitals, long-term care facilities, community, quarantine facilities, nursing homes, and cruise ships. Populations include immunocompromised patients, health care workers, patients with specific diseases, and general or specific community groups. Funding sources range from national research foundations, government agencies, philanthropic organizations, and some studies report no external funding or not reported.

Navigate back to Table 2..

### Table 3. Long description

The table contains seven columns. The first column lists study names, including Alshukairi 2021, Avanzato 2021, Aydillo 2020, Baang 2021, Basheer 2021, Choi et al., Decker et al., Garcia-Knight et al., Gniazdowski 2021, Guetl et al., Han 2021, Jung et al., Kang et al., Ke 2021, Kim 2022a, Kim 2022b, Kujavski 2020, Laferl 2020, Lang 2020, Leitão 2021, Leung 2022, Lin et al., Lu 2020, Luna-Muschi 2022, Mancon 2022, McCormick 2023, Mendes-Correa 2021, Mileto 2021, Monrad et al., Murata 2021, Nissen 2021, Niyonkuru 2021, Nomura et al., Pedro 2021, Pérez-Lago 2021, Pickering 2021, Rajakumar 2021, Sepulcri et al., Siedner et al., Singh et al., Spinicci 2022, Sung 2022, Tarhini 2021, Thornton 2022, Tobolowski 2022, Truong 2021, Weigang 2021, Williamson 2021, Zahn 2021, Zupin 2022. The next five columns are: ‘Were the criteria for diagnosing a case clearly reported and appropriate’, ‘Was the reporting of patient/population characteristics adequate’, ‘Were the methods used to obtain R T dash P C R results replicable, generalizable and appropriate’, ‘Were the methods used to obtain viral culture results replicable and appropriate’, and ‘Was the study period, including follow up, sufficient’. Each cell in these columns contains either Yes, No, or Unclear. The final column, ‘Notes’, provides detailed comments for each study, such as missing data on R T dash P C R controls, lack of information on viral culture methods, Ct cutoff values, and limitations in symptom reporting. Some studies, such as Choi et al., Lin et al., Rajakumar 2021, Kim 2022b, and Weigang 2021, meet all criteria with Yes in all columns. Many studies have Unclear or No in one or more columns, with frequent notes about missing Ct cutoff, lack of controls, or insufficient reporting of clinical symptoms or culture methods.

Navigate back to Table 3..

### Table 4. Long description

From the top row, the table has two columns labeled Characteristic and Value. The first row lists Number of patients with N equals 381. The second row lists Number of studies with N equals 39. The third row lists Number of observations per patient with Median 3 and I Q R 2 to 7. The fourth row lists Length of time following first R T dash P C R with Median 5 and I Q R 0 to 11. The fifth row lists Number and percent of patients who were culture-positive at first test with 148 and 38.8 percent. The sixth row lists Number and percent of patients who were symptomatic at first test with 253 and 66.4 percent.

Navigate back to Table 4..

### Table 5. Long description

The table presents three main groups: Culture, Time since the first PCR, and COVID 19 treatments. The first row gives the overall average at reference levels with a cycle threshold value of 30.8 and a 95 percent confidence interval of 27.8 to 33.8. Under Culture, the reference level is Negative. Positive culture is associated with a change in cycle threshold of minus 6.55 with a 95 percent confidence interval of minus 7.83 to minus 5.26 and a P value less than 0.001. For Time since the first PCR, less than 7 days is the reference. At 7 to 13 days, the change is plus 2.37 with a 95 percent confidence interval of 0.622 to 4.080. At 14 to 28 days, the change is plus 4.06 with a 95 percent confidence interval of 2.04 to 6.06. At 28 days or more, the change is plus 5.96 with a 95 percent confidence interval of 3.18 to 8.69. The P value for this group is less than 0.001. For COVID 19 treatments, ‘No data on treatments’ is the reference. ‘No mention’ shows a change of 0.45 with a 95 percent confidence interval of minus 2.07 to 2.98. ‘Yes’ shows a change of minus 5.51 with a 95 percent confidence interval of minus 8.15 to minus 2.00. The P value for this group is less than 0.001.

Navigate back to Table 5..

### Table 6. Long description

The table has three columns labeled Characteristic, Odds ratio with approximate 95 percent confidence interval, and P value. The first row lists CT value with odds ratio 0.84, confidence interval 0.804 to 0.877, and P value less than 0.0001. The next section is Time since the first PCR, subdivided into less than 7 days (reference level), 7 to 13 days with odds ratio 0.54 and confidence interval 0.267 to 1.11, 14 to 28 days with odds ratio 0.286 and confidence interval 0.139 to 0.589, and 28 days or more with odds ratio 0.635 and confidence interval 0.184 to 2.19. The P value for this section is 0.005. The final section is Cancer, subdivided into No clinical data reported (reference level), No cancer with odds ratio 1.23 and confidence interval 0.691 to 2.19, and Cancer with odds ratio 5.59 and confidence interval 2.34 to 13.3. The P value for this section is 0.0002.

Navigate back to Table 6..
