# Supplementary material for: Serial cycle threshold to assess the infectious potential of SARS-CoV-2: A systematic review
Source: Epidemiol Infect. 2026 May 6;154:e89. doi: 10.1017/S0950268826101484 (PMC13366375; doi:10.1017/S0950268826101484)
Supplement: Rosca et al. supplementary material [file S0950268826101484sup001.zip › Appendix 1. METHODOLOGICAL QUALITY ASSESSMENT.docx]

**METHODOLOGICAL QUALITY ASSESSMENT**

Quality assessment

We assessed the quality of the included studies according to modified QUADAS criteria that we have used previously. [3, 10]

1. Were the criteria for diagnosing a case reported and appropriate?

2. Was reporting patient/population characteristics, including clinical symptoms, treatments and outcomes, adequate?

3. Was the study period, including follow-up, sufficient to adequately assess any potential relationship between viral burden measures and the likelihood of producing replication-competent virus and the rise in neutralising antibodies?

4. Were the methods used to obtain RT-PCR results replicable, generalizable and appropriate? We considered that each study should use internal standards to establish the relationship between their Ct values and the target gene copy number.

5. Were the methods used to obtain viral culture results replicable and appropriate?

We considered the methods used should, at a minimum, include a description of specimen sampling and management, preparation, media and cell line used, exclusion of contamination or co-infection (use of suitable controls and appropriate antibacterials and antimycotics and possible use of gene sequencing if available), and results of the inspection of culture.
