# Supplementary material for: Serial cycle threshold to assess the infectious potential of SARS-CoV-2: A systematic review
Source: Epidemiol Infect. 2026 May 6;154:e89. doi: 10.1017/S0950268826101484 (PMC13366375; doi:10.1017/S0950268826101484)
Supplement: Rosca et al. supplementary material [file S0950268826101484sup001.zip › Appendix 2. List of excluded studies.docx]

Appendix 2. Supplementary data. A list of studies excluded after reading the full text, with reasons.

|  | Study reference | Reason for exclusion |
| --- | --- | --- |
|  | Acharya CB, Schrom J, Mitchell AM, et al. No Significant Difference in Viral Load Between Vaccinated and Unvaccinated, Asymptomatic and Symptomatic Groups When Infected with SARS-CoV-2 Delta Variant. medRxiv 2021.09.28.21264262; doi: https://doi.org/10.1101/2021.09.28.21264262 | No viral cultures |
|  | Ahn JY, An S, Sohn Y, Cho Y, Hyun JH, Baek YJ, Kim MH, Jeong SJ, Kim JH, Ku NS, Yeom JS, Smith DM, Lee H, Yong D, Lee YJ, Kim JW, Kim HR, Hwang J, Choi JY. Environmental contamination in the isolation rooms of COVID-19 patients with severe pneumonia requiring mechanical ventilation or high-flow oxygen therapy. J Hosp Infect. 2020 Nov;106(3):570-576. doi: 10.1016/j.jhin.2020.08.014. | No respiratory samples |
|  | Alshukairi AN, El-Kafrawy SA, Dada A, et al. Re-infection with a different SARS-CoV-2 clade and prolonged viral shedding in a hematopoietic stem cell transplantation patient. Int J Infect Dis. 2021 Sep;110:267-271. doi: 10.1016/j.ijid.2021.07.036. | No data on serial Cts |
|  | Andersson MI, Arancibia-Carcamo CV, Auckland K et al. SARS-CoV-2 RNA detected in blood products from patients with COVID-19 is not associated with infectious virus [version 1; peer review: 1 approved with reservations]. Wellcome Open Res 2020, 5:181 (https://doi.org/10.12688/wellcomeopenres.16002.1) | No respiratory samples |
|  | Antar AAR, Yu T, Pisanic N, et al. Delayed Rise of Oral Fluid Antibodies, Elevated BMI, and Absence of Early Fever Correlate With Longer Time to SARS-CoV-2 RNA Clearance in a Longitudinally Sampled Cohort of COVID-19 Outpatients. Open Forum Infect Dis. 2021 Apr 16;8(6):ofab195. doi: 10.1093/ofid/ofab195. | Aggregated data |
|  | Antar AA.; Yu T, Azamfirei R, et al. Host factors associated with persistent SARS-CoV-2 viral RNA in COVID-19 outpatients. Topics in Antiviral Medicine ; 29(1):68, 2021 | Abstract of Antar et al. doi: 10.1093/ofid/ofab195. |
|  | Arons MM, Hatfield KM, Reddy SC, et al. Presymptomatic SARS-CoV-2 Infections and Transmission in a Skilled Nursing Facility. N Engl J Med. 2020 May 28;382(22):2081-2090. doi: 10.1056/NEJMoa2008457. | data on serial Cts cannot be extracted at an individual level |
|  | Auewarakul P, Sirihongthong T, Boonarkart C, et al. Dynamics of SAR-CoV2 Viral Load Decline and Antibody Responses in COVID-19 Patients, 18 February 2022, PREPRINT (Version 1) available at Research Square [https://doi.org/10.21203/rs.3.rs-1172677/v1] | Aggregated data |
|  | Atarere J, Zhou Z, Turcinovic J, et al. Performance of Rapid Diagnostic Testing at Days 4-6 from Diagnosis: Implications for Discharge from Isolation on a University Campus, Open Forum Infectious Diseases, Volume 9, Issue Supplement_2, December 2022, ofac492.391, https://doi.org/10.1093/ofid/ofac492.391 | Aggregated data. Meeting abstract |
|  | Bal A, Brengel-Pesce K, Gaymard A, et al. Clinical and laboratory characteristics of symptomatic healthcare workers with suspected COVID-19: a prospective cohort study. Sci Rep. 2021 Jul 22;11(1):14977. doi: 10.1038/s41598-021-93828-y. Erratum in: Sci Rep. 2021 Sep 23;11(1):19317. | Aggregated data |
|  | Bal A, Brengel-Pesce K, Gaymard A, et al. Clinical and microbiological assessments of COVID-19 in healthcare workers: a prospective longitudinal study. medRxiv 2020.11.04.20225862; doi: <https://doi.org/10.1101/2020.11.04.20225862> | Preprint of doi: 10.1038/s41598-021-93828-y |
|  | Bardossy AC, Korhonen L, Schatzman S, et al. Clinical Course of SARS-CoV-2 Infection in Adults with ESKD Receiving Outpatient Hemodialysis. Kidney360. 2021 Sep 22;2(12):1917-1927. doi: 10.34067/KID.0004372021. | Aggregated data |
|  | Basile K, McPhie K, Carter I, et al. Cell-based Culture Informs Infectivity and Safe De-Isolation Assessments in Patients with Coronavirus Disease 2019. Clin Infect Dis. 2021 Nov 2;73(9):e2952-e2959. doi: 10.1093/cid/ciaa1579. | Aggregated data; no serial Cts |
|  | Benotmane I, Risch S, Doderer-Lang C, Caillard S, Fafi-Kremer S. Long-term shedding of viable SARS-CoV-2 in kidney transplant recipients with COVID-19. Am J Transplant. 2021 Aug;21(8):2871-2875. doi: 10.1111/ajt.16636. | Aggregated data |
|  | Beran A, Zink E, Mhanna M, et al. Transmissibility and viral replication of SARS-COV-2 in immunocompromised patients. J Med Virol. 2021 Jul;93(7):4156-4160. doi: 10.1002/jmv.26970. | Review |
|  | Berengua C, López M, Esteban M, et al. Viral culture and immunofluorescence for the detection of SARS-CoV-2 infectivity in RT-PCR positive respiratory samples. J Clin Virol. 2022 Jul;152:105167. doi: 10.1016/j.jcv.2022.105167. | Aggregated data |
|  | Bhat V, Chavan P, Khattry N, Gupta S. Dynamics of viral RNA load, virus culture, seroconversion & infectivity in COVID-19 patients: Implications on isolation policy. Indian J Med Res. 2021 May&Jun;153(5&6):585-590. doi: 10.4103/ijmr.IJMR_3564_20. | Review |
|  | Binder RA, Alarja NA, et al. Environmental and Aerosolized Severe Acute Respiratory Syndrome Coronavirus 2 Among Hospitalized Coronavirus Disease 2019 Patients. J Infect Dis. 2020 Nov 9;222(11):1798-1806. doi: 10.1093/infdis/jiaa575. | Environmental study |
|  | Bonenfant G, Deyoe J, Wong T, et al. Surveillance and correlation of SARS-CoV-2 viral RNA, antigen, virus isolation, and self-reported symptoms in a longitudinal study with daily sampling. medRxiv 2021.12.23.21268319; doi: https://doi.org/10.1101/2021.12.23.21268319 | Aggregated data |
|  | Bonenfant G, Deyoe JE, Wong T, et al. Surveillance and Correlation of Severe Acute Respiratory Syndrome Coronavirus 2 Viral RNA, Antigen, Virus Isolation, and Self-Reported Symptoms in a Longitudinal Study With Daily Sampling. Clin Infect Dis. 2022 Nov 14;75(10):1698-1705. doi: 10.1093/cid/ciac282. | Aggregated data. Also published on MedRxiv (https://doi.org/10.1101/2021.12.23.21268319 ) |
|  | Borges V, Isidro J, Cunha M, et al. Long-Term Evolution of SARS-CoV-2 in an Immunocompromised Patient with Non-Hodgkin Lymphoma. mSphere. 2021 Aug 25;6(4):e0024421. doi: 10.1128/mSphere.00244-21. | No data on Ct |
|  | Brown CS, Clare K, Chand M, et al. Snapshot PCR surveillance for SARS-CoV-2 in hospital staff in England. J Infect. 2020 Sep;81(3):427-434. doi: 10.1016/j.jinf.2020.06.069. | no serial Cts |
|  | Borczuk AC, Salvatore SP, Seshan SV, et al. COVID-19 pulmonary pathology: a multi-institutional autopsy cohort from Italy and New York City. Mod Pathol. 2020 Nov;33(11):2156-2168. doi: 10.1038/s41379-020-00661-1. | post-mortem study |
|  | Boucau J, Marino C, Regan J, et al. Duration of Shedding of Culturable Virus in SARS-CoV-2 Omicron (BA.1) Infection. N Engl J Med. 2022 Jul 21;387(3):275-277. doi: 10.1056/NEJMc2202092. | Aggregated data |
|  | Bouton TC, Atarere J, Turcinovic J, et al. Viral Dynamics of Omicron and Delta Severe Acute Respiratory Syndrome Coronavirus 2 (SARS-CoV-2) Variants With Implications for Timing of Release from Isolation: A Longitudinal Cohort Study. Clin Infect Dis. 2023 Feb 8;76(3):e227-e233. doi: 10.1093/cid/ciac510. | Aggregated data |
|  | Bullard J, Dust K, Funk D, et al. Predicting Infectious Severe Acute Respiratory Syndrome Coronavirus 2 From Diagnostic Samples. Clin Infect Dis. 2020 Dec 17;71(10):2663-2666. doi: 10.1093/cid/ciaa638. | no serial Cts |
|  | Caccuri F, Zani A, Messali S, et al. A persistently replicating SARS-CoV-2 variant derived from an asymptomatic individual. J Transl Med. 2020 Sep 23;18(1):362. doi: 10.1186/s12967-020-02535-1. | No serial Cts |
|  | Carmagnola D, Pellegrini G, Canciani E, et al. Saliva Molecular Testing for SARS-CoV-2 Surveillance in Two Italian Primary Schools. Children (Basel). 2021 Jun 24;8(7):544. doi: 10.3390/children8070544. | No viral cultures |
|  | Casarola G, D'Abbondanza M, Curcio R, et al. Efficacy of convalescent plasma therapy in immunocompromised patients with COVID-19: A case report. Clin Infect Pract. 2021 Nov;12:100096. doi: 10.1016/j.clinpr.2021.100096. | No data on Ct |
|  | Cele S, Karim F, Lustig G, et al. SARS-CoV-2 prolonged infection during advanced HIV disease evolves extensive immune escape. Cell Host Microbe. 2022 Feb 9;30(2):154-162.e5. doi: 10.1016/j.chom.2022.01.005. | Neutralization study |
|  | Ceulemans LJ, Khan M, Yoo SJ, et al. Persistence of SARS-CoV-2 RNA in lung tissue after mild COVID-19. Lancet Respir Med. 2021 Aug;9(8):e78-e79. doi: 10.1016/S2213-2600(21)00240-X. | Biopsy study |
|  | Charmetant X, Espi M, Benotmane I, et al. Comparison of infected and vaccinated transplant recipients highlights the role of Tfh and neutralizing IgG in COVID-19 protection. medRxiv 2021.07.22.21260852; doi: https://doi.org/10.1101/2021.07.22.21260852 | No viral cutures |
|  | Chen RE, Gorman MJ, Zhu DY, et al. Reduced antibody activity against SARS-CoV-2 B.1.617.2 Delta virus in serum of mRNA-vaccinated patients receiving TNF-α inhibitors. medRxiv 2021.09.28.21264250; doi: https://doi.org/10.1101/2021.09.28.21264250 | Immunological study |
|  | Christofferson RC, Giovanni JE, Koumans EH, et al. A Systematic Review of Prolonged SARS-CoV-2 Shedding in Immunocompromised Persons. Influenza Other Respir Viruses. 2025;19(5):e70121. doi: 10.1111/irv.70121. Erratum in: Influenza Other Respir Viruses. 2025 Jun;19(6):e70127. doi: 10.1111/irv.70127. | Systematic review |
|  | Cimrman Š, Macková L, Král V, Bartoš H, Stiborová I, Dlouhý P. The duration of SARS-CoV-2 shedding in patients recovering from COVID-19. Epidemiol Mikrobiol Imunol. 2020 Summer;69(3):148-151. | No viral cultures |
|  | Chu VT, Schwartz NG, Donnelly MAP, et al. Comparison of Home Antigen Testing With RT-PCR and Viral Culture During the Course of SARS-CoV-2 Infection. JAMA Intern Med. 2022 Jul 1;182(7):701-709. doi: 10.1001/jamainternmed.2022.1827. Erratum in: JAMA Intern Med. 2023 Jul 1;183(7):748. | Data cannot be extracted at an individual level |
|  | Clark SA, Clark LE, Pan J, et al. SARS-CoV-2 evolution in an immunocompromised host reveals shared neutralization escape mechanisms. Cell. 2021 May 13;184(10):2605-2617.e18. doi: 10.1016/j.cell.2021.03.027. | No data on Ct |
|  | Drain PK, Dalmat RR, Hao L, et al. Duration of viral infectiousness and correlation with symptoms and diagnostic testing in non-hospitalized adults during acute SARS-CoV-2 infection: A longitudinal cohort study. J Clin Virol. 2023 Apr;161:105420. doi: 10.1016/j.jcv.2023.105420. | Individual data cannot be extracted from supplemental figure 3 |
|  | Drancourt M, Cortaredona S, Melenotte C, et al. SARS-CoV-2 Persistent Viral Shedding in the Context of Hydroxychloroquine-Azithromycin Treatment. Viruses. 2021 May 12;13(5):890. doi: 10.3390/v13050890. | Data cannot be extracted at an individual level |
|  | Dergham J, Delerce J, Bedotto M, La Scola B, Moal V. Isolation of Viable SARS-CoV-2 Virus from Feces of an Immunocompromised Patient Suggesting a Possible Fecal Mode of Transmission. J Clin Med. 2021 Jun 18;10(12):2696. doi: 10.3390/jcm10122696. | Not respiratory samples |
|  | Dzieciolowska S, Charest H, Roy T, et al. Timing and Predictors of Loss of Infectivity Among Healthcare Workers With Mild Primary and Recurrent COVID-19: A Prospective Observational Cohort Study. Clin Infect Dis. 2024;78(3):613-624. doi: 10.1093/cid/ciad535. | Data cannot be extracted at an individual level |
|  | Fisman DN, Tuite AR. Evaluation of the relative virulence of novel SARS-CoV-2 variants: a retrospective cohort study in Ontario, Canada. CMAJ. 2021 Oct 25;193(42):E1619-E1625. doi: 10.1503/cmaj.211248. Epub 2021 Oct 4. | No viral cultures |
|  | Folgueira MD, Luczkowiak J, Lasala F, et al. Prolonged SARS-CoV-2 cell culture replication in respiratory samples from patients with severe COVID-19. Clin Microbiol Infect. 2021 Jun;27(6):886-891. doi: 10.1016/j.cmi.2021.02.014. | Data cannot be extracted at an individual level |
|  | Fontana LM, Villamagna AH, Sikka MK, McGregor JC. Understanding viral shedding of severe acute respiratory coronavirus virus 2 (SARS-CoV-2): Review of current literature. Infect Control Hosp Epidemiol. 2021 Jun;42(6):659-668. doi: 10.1017/ice.2020.1273. | Review |
|  | Ford L, Lee C, Pray IW, et al. Epidemiologic Characteristics Associated With Severe Acute Respiratory Syndrome Coronavirus 2 (SARS-CoV-2) Antigen-Based Test Results, Real-Time Reverse Transcription Polymerase Chain Reaction (rRT-PCR) Cycle Threshold Values, Subgenomic RNA, and Viral Culture Results From University Testing. Clin Infect Dis. 2021 Sep 15;73(6):e1348-e1355. doi: 10.1093/cid/ciab303. | No serial Cts |
|  | Frampton D, Rampling T, Cross A, et al. Genomic characteristics and clinical effect of the emergent SARS-CoV-2 B.1.1.7 lineage in London, UK: a whole-genome sequencing and hospital-based cohort study. Lancet Infect Dis. 2021 Sep;21(9):1246-1256. doi: 10.1016/S1473-3099(21)00170-5. | No viral cultures |
|  | Francis R, Le Bideau M, Jardot P, et al. High-speed large-scale automated isolation of SARS-CoV-2 from clinical samples using miniaturized co-culture coupled to high-content screening. Clin Microbiol Infect. 2021 Jan;27(1):128.e1-128.e7. doi: 10.1016/j.cmi.2020.09.018. | No serial Cts |
|  | Funk DJ, Bullard J, Lother S, et al. Persistence of live virus in critically ill patients infected with SARS-COV-2: a prospective observational study. Crit Care. 2022 Jan 4;26(1):10. doi: 10.1186/s13054-021-03884-z. | Aggregated data |
|  | Gable P, Huang JY, Gilbert SE, et al. A Comparison of Less Invasive Severe Acute Respiratory Syndrome Coronavirus 2 (SARS-CoV-2) Diagnostic Specimens in Nursing Home Residents-Arkansas, June-August 2020. Clin Infect Dis. 2021 Jul 15;73(Suppl 1):S58-S64. doi: 10.1093/cid/ciab310. | Aggregated data |
|  | Gaspar-Rodríguez A, Padilla-González A, Rivera-Toledo E. Coronavirus persistence in human respiratory tract and cell culture: An overview. Braz J Infect Dis. 2021 Sep-Oct;25(5):101632. doi: 10.1016/j.bjid.2021.101632. | Review |
|  | Gilbert M, Atarere J, Turcinovic J,et al. Time from last COVID-19 vaccination’s impact on rapidity of viral culture conversion following SARS-CoV-2 infection: a prospective cohort study, Open Forum Infectious Diseases, Volume 9, Issue Supplement_2, December 2022, ofac492.088, https://doi.org/10.1093/ofid/ofac492.088 | Aggregated data. Meeting abstract |
|  | Glans H, Gredmark-Russ S, Olausson M, et al. Shedding of infectious SARS-CoV-2 by hospitalized COVID-19 patients in relation to serum antibody responses. BMC Infect Dis. 2021 May 27;21(1):494. doi: 10.1186/s12879-021-06202-8. | No data on serial Ct |
|  | Gohli J, Anderson AM, Brantsaeter AB, Bøifot KO, Grub C, Hadley CL, Lind A, Pettersen ES, Søraas AVL, Dybwad M. Dispersion of SARS-CoV-2 in air surrounding COVID-19-infected individuals with mild symptoms. Indoor Air. 2022 Feb;32(2):e13001. doi: 10.1111/ina.13001. | Viral cultures were from environmental samples |
|  | Hagan LM, McCormick DW, Lee C, et al. Outbreak of SARS-CoV-2 B.1.617.2 (Delta) Variant Infections Among Incarcerated Persons in a Federal Prison - Texas, July-August 2021. MMWR Morb Mortal Wkly Rep. 2021 Sep 24;70(38):1349-1354. doi: 10.15585/mmwr.mm7038e3. | Aggregated data |
|  | Hawken SE, Sellers SA, Smedberg JR, et al. Longitudinal SARS-CoV-2 Testing among the Unvaccinated Is Punctuated by Intermittent Positivity and Variable Rates of Increasing Cycle Threshold Values. Microbiol Spectr. 2022 Apr 27;10(2):e0271521. doi: 10.1128/spectrum.02715-21. | No viral cultures |
|  | Hay JA, Kissler SM, Fauver JR, et al. Viral dynamics and duration of PCR positivity of the SARS-CoV-2 Omicron variant. medRxiv 2022.01.13.22269257; doi: https://doi.org/10.1101/2022.01.13.22269257 | No viral cultures |
|  | Hensley MK, Bain WG, Jacobs J, et al. Intractable Coronavirus Disease 2019 (COVID-19) and Prolonged Severe Acute Respiratory Syndrome Coronavirus 2 (SARS-CoV-2) Replication in a Chimeric Antigen Receptor-Modified T-Cell Therapy Recipient: A Case Study. Clin Infect Dis. 2021 Aug 2;73(3):e815-e821. doi: 10.1093/cid/ciab072. | No serial Cts for respiratory samples |
|  | Huang CG, Lee KM, Hsiao MJ, et al. Culture-Based Virus Isolation To Evaluate Potential Infectivity of Clinical Specimens Tested for COVID-19. J Clin Microbiol. 2020 Jul 23;58(8):e01068-20. doi: 10.1128/JCM.01068-20. | No serial PCR |
|  | Huang YC, Tu HC, Kuo HY, et al. Outbreak investigation in a COVID-19 designated hospital: The combination of phylogenetic analysis and field epidemiology study suggesting airborne transmission. J Microbiol Immunol Infect. 2023 Jun;56(3):547-557. doi: 10.1016/j.jmii.2023.01.003. | No viral cultures |
|  | Jaumdally S, Tomasicchio M, Pooran A, et al. Frequency, kinetics and determinants of viable SARS-CoV-2 in bioaerosols from ambulatory COVID-19 patients infected with the Beta, Delta or Omicron variants. Nat Commun. 2024;15(1):2003. doi: 10.1038/s41467-024-45400-1. | Aggregated data |
|  | Jeong HW, Kim SM, Kim HS, et al. Viable SARS-CoV-2 in various specimens from COVID-19 patients. Clin Microbiol Infect. 2020 Nov;26(11):1520-1524. doi: 10.1016/j.cmi.2020.07.020. 2020 Jul 23. PMID: 32711057; PMCID: PMC7375961. | No serial Cts |
|  | Jiwani RA, Mao Y, Pona A, et al. Discontinuation of Transmission Precautions for COVID-19 Patients: Polymerase Chain Reaction Diagnostics, Patient Delays, and Cycle Threshold Values. Infect Dis Clin Pract (Baltim Md). 2021 Sep;29(5):e287-e293. doi: 10.1097/IPC.0000000000001005. | No viral cultures |
|  | Jung J, Kim JY, Park H, et al. Transmission and Infectious SARS-CoV-2 Shedding Kinetics in Vaccinated and Unvaccinated Individuals. JAMA Netw Open. 2022 May 2;5(5):e2213606. doi: 10.1001/jamanetworkopen.2022.13606. | Aggregated data |
|  | Katz MJ, Reeves M, Harris TG, et al. Kinetics of SARS-CoV-2 Shedding in Nursing Home Residents and Staff. J Am Geriatr Soc. 2025. doi: 10.1111/jgs.19499. | Data cannot be extracted from figure |
|  | Keske Ş, Güney-Esken G, Vatansever C, et al. Duration of infectious shedding of SARS-CoV-2 Omicron variant and its relation with symptoms. Clin Microbiol Infect. 2023 Feb;29(2):221-224. doi: 10.1016/j.cmi.2022.07.009. | Aggregated data |
|  | Khatamzas E, Rehn A, Muenchhoff M, et al. Emergence of multiple SARS-CoV-2 mutations in an immunocompromised host. medRxiv 2021.01.10.20248871; doi: https://doi.org/10.1101/2021.01.10.20248871 | Data cannot be extracted from figure |
|  | Kim JM, Kim HM, Lee EJ, et al. Detection and Isolation of SARS-CoV-2 in Serum, Urine, and Stool Specimens of COVID-19 Patients from the Republic of Korea. Osong Public Health Res Perspect. 2020 Jun;11(3):112-117. doi: 10.24171/j.phrp.2020.11.3.02. | No viral cultures from respiratory specimens |
|  | Klein J, Brito AF, Trubin P, et al. Longitudinal Immune Profiling of a Severe Acute Respiratory Syndrome Coronavirus 2 Reinfection in a Solid Organ Transplant Recipient. J Infect Dis. 2022 Feb 1;225(3):374-384. doi: 10.1093/infdis/jiab553. | No culture data |
|  | Kemp SA, Collier DA, Datir RP,et al. SARS-CoV-2 evolution during treatment of chronic infection. Nature. 2021 Apr;592(7853):277-282. doi: 10.1038/s41586-021-03291-y. Epub 2021 Feb 5. Erratum in: Nature. 2022 Aug;608(7922):E23. | No viral cultures |
|  | Kociolek LK, Muller WJ, Yee R, et al. Comparison of Upper Respiratory Viral Load Distributions in Asymptomatic and Symptomatic Children Diagnosed with SARS-CoV-2 Infection in Pediatric Hospital Testing Programs. J Clin Microbiol. 2020 Dec 17;59(1):e02593-20. doi: 10.1128/JCM.02593-20. | No viral cultures |
|  | Koff AG, Laurent-Rolle M, Hsu JC, Malinis M. Prolonged incubation of severe acute respiratory syndrome coronavirus 2 (SARS-CoV-2) in a patient on rituximab therapy. Infect Control Hosp Epidemiol. 2021 Oct;42(10):1286-1288. doi: 10.1017/ice.2020.1239. | No serial Cts for the samples used for viral culture. |
|  | Korean CDC: Prevention. KCfDCa. Findings from investigation and analysis of re-positive cases” 2020-05-19 ~ 2020-12-31. <https://www.cdc.go.kr/board/board.es?mid=&bid=0030&act=view&list_no=367267&nPage=1> | aggregated data |
|  | Lacson E Jr, Weiner D, Majchrzak K, et al. Prolonged Live SARS-CoV-2 Shedding in a Maintenance Dialysis Patient. Kidney Med. 2021 Mar-Apr;3(2):309-311. doi: 10.1016/j.xkme.2020.12.001. | No data on Ct |
|  | L'Huillier AG, Torriani G, Pigny F, Kaiser L, Eckerle I. Culture-Competent SARS-CoV-2 in Nasopharynx of Symptomatic Neonates, Children, and Adolescents. Emerg Infect Dis. 2020 Oct;26(10):2494-2497. doi: 10.3201/eid2610.202403. | no serial Cts |
|  | La Scola B, Le Bideau M, Andreani J, et al. Viral RNA load as determined by cell culture as a management tool for discharge of SARS-CoV-2 patients from infectious disease wards. Eur J Clin Microbiol Infect Dis. 2020 Jun;39(6):1059-1061. doi: 10.1007/s10096-020-03913-9. | aggregated data |
|  | Ladhani SN, Chow JY, Janarthanan R, et al. Investigation of SARS-CoV-2 outbreaks in six care homes in London, April 2020. EClinicalMedicine. 2020 Sep; 26:100533. doi: 10.1016/j.eclinm.2020.100533. | no serial Cts; aggregated data |
|  | Leal J, O'Grady HM, Armstrong L, et al. Patient and ward related risk factors in a multi-ward nosocomial outbreak of COVID-19: Outbreak investigation and matched case-control study. Antimicrob Resist Infect Control. 2023 Mar 22;12(1):21. doi: 10.1186/s13756-023-01215-1. | Aggregated data |
|  | Longtin Y, Parkes LO, Charest H, et al. Persistence of infectivity in elderly individuals diagnosed with severe acute respiratory coronavirus virus 2 (SARS-CoV-2) infection 10 days after onset of symptoms: A cross-sectional study. Infect Control Hosp Epidemiol. 2021 Dec 6:1-4. doi: 10.1017/ice.2021.502. | Aggregated data |
|  | Lutgring JD, Tobolowsky FA, Hatfield KM, et al. Evaluating the Presence of Replication-Competent Severe Acute Respiratory Syndrome Coronavirus 2 (SARS-CoV-2) From Nursing Home Residents With Persistently Positive Reverse Transcription Polymerase Chain Reaction (RT-PCR) Results. Clin Infect Dis. 2022 Feb 11;74(3):525-528. doi: 10.1093/cid/ciab436. | Aggregated data |
|  | Mack CD, DiFiori J, Tai CG, et al. SARS-CoV-2 Transmission Risk Among National Basketball Association Players, Staff, and Vendors Exposed to Individuals With Positive Test Results After COVID-19 Recovery During the 2020 Regular and Postseason. JAMA Intern Med. 2021 Jul 1;181(7):960-966. doi: 10.1001/jamainternmed.2021.2114. | No viral cultures |
|  | Manabe YC, Reuland C, Yu T, et al. Variability of Salivary and Nasal Specimens for SARS-CoV-2 Detection. medRxiv 2020.10.07.20208520; doi: https://doi.org/10.1101/2020.10.07.20208520 | Aggregated data |
|  | Manzulli V, Scioscia G, Giganti G, et al. Real Time PCR and Culture-Based Virus Isolation Test in Clinically Recovered Patients: Is the Subject Still Infectious for SARS-CoV2? J Clin Med. 2021 Jan 15;10(2):309. doi: 10.3390/jcm10020309. | Aggregated data |
|  | Mathur S, Davidson MC, Anglin K, et al. Evaluation of Severe Acute Respiratory Syndrome Coronavirus 2 Nucleocapsid Antigen in the Blood as a Diagnostic Test for Infection and Infectious Viral Shedding. Open Forum Infect Dis. 2022 Oct 22;9(11):ofac563. doi: 10.1093/ofid/ofac563. | Aggregated data |
|  | McCormick DW, Konkle SL, Magleby R, et al. SARS-CoV-2 infection risk among vaccinated and unvaccinated household members during the Alpha variant surge - Denver, Colorado, and San Diego, California, January-April 2021. Vaccine. 2022 Aug 5;40(33):4845-4855. doi: 10.1016/j.vaccine.2022.06.066. | Aggregated data |
|  | McKay SL, Tobolowsky FA, Moritz ED, et al. Performance Evaluation of Serial SARS-CoV-2 Rapid Antigen Testing During a Nursing Home Outbreak. Ann Intern Med. 2021 Jul;174(7):945-951. doi: 10.7326/M21-0422. | Aggregated data |
|  | Mellis AM, Meece JK, Halasa NB, et al. SARS-CoV-2 Virus Dynamics in Recently Infected People-Data From a Household Transmission Study. J Infect Dis. 2022 Nov 11;226(10):1699-1703. doi: 10.1093/infdis/jiac180. | Aggregated data |
|  | Mendes Correa MC, Leal FE, Villas Boas LS, et al. Prolonged presence of replication-competent SARS-CoV-2 in mildly symptomatic individuals: A report of two cases. J Med Virol. 2021 Sep;93(9):5603-5607. doi: 10.1002/jmv.27021 | No data on Cts |
|  | Mollan KR, Eron JJ, Krajewski TJ, et al. Infectious Severe Acute Respiratory Syndrome Coronavirus 2 (SARS-CoV-2) Virus in Symptomatic Coronavirus Disease 2019 (COVID-19) Outpatients: Host, Disease, and Viral Correlates. Clin Infect Dis. 2022 Aug 24;75(1):e1028-e1036. doi: 10.1093/cid/ciab968. | No serial Cts |
|  | Morel A, Imbeaud S, Scemla A, et al. Severe relapse of SARS-CoV-2 infection in a kidney transplant recipient with negative nasopharyngeal SARS-CoV-2 RT-PCR after rituximab. Am J Transplant. 2022 Aug;22(8):2099-2103. doi: 10.1111/ajt.17000. | Viral culture is from BAL. Serial Cts are for NP swab. Date of BAL is approximate |
|  | Nemudryi A, Nemudraia A, Wiegand T, et al. SARS-CoV-2 genomic surveillance identifies naturally occurring truncation of ORF7a that limits immune suppression. Cell Rep. 2021 Jun 1;35(9):109197. doi: 10.1016/j.celrep.2021.109197. | Genomic study |
|  | Park S, Lim SY, Kim JY, et al. Clinical and Virological Characteristics of Severe Acute Respiratory Syndrome Coronavirus 2 (SARS-CoV-2) B.1.617.2 (Delta) Variant: A Prospective Cohort Study. Clin Infect Dis. 2022 Aug 24;75(1):e27-e34. doi: 10.1093/cid/ciac239. | Aggregated data |
|  | Perera RAPM, Tso E, Tsang OTY, et al. SARS-CoV-2 Virus Culture and Subgenomic RNA for Respiratory Specimens from Patients with Mild Coronavirus Disease. Emerg Infect Dis. 2020 Nov;26(11):2701-2704. doi: 10.3201/eid2611.203219. | Aggregated data |
|  | Perera RAPM, Tso E, Tsang OTY, et al. SARS-CoV-2 virus culture from the upper respiratory tract: Correlation with viral load, subgenomic viral RNA and duration of illness. medRxiv 2020.07.08.20148783; doi: https://doi.org/10.1101/2020.07.08.20148783 | Preprint of doi: 10.3201/eid2611.203219. |
|  | Phuphuakrat A, Pasomsub E, Srichatrapimuk S, et al. Detectable Duration of Viable SARS-CoV-2, Total and Subgenomic SARS-CoV-2 RNA in Noncritically Ill COVID-19 Patients: a Prospective Cohort Study. Microbiol Spectr. 2022 Jun 29;10(3):e0050322. doi: 10.1128/spectrum.00503-22. | Data cannot be extracted at individual level |
|  | Puchinger K, Castelletti N, Rubio-Acero R, et al. The interplay of viral loads, clinical presentation, and serological responses in SARS-CoV-2 - Results from a prospective cohort of outpatient COVID-19 cases. Virology. 2022 Apr;569:37-43. doi: 10.1016/j.virol.2022.02.002. | Data cannot be extracted at individual level |
|  | Puhach O, Adea K, Hulo N, et al. Infectious viral load in unvaccinated and vaccinated patients infected with SARS-CoV-2 WT, Delta and Omicron. medRxiv 2022.01.10.22269010; doi: https://doi.org/10.1101/2022.01.10.22269010 | Aggregated data |
|  | Purpura LJ, Chang M, Annavajhala MK, et al. Prolonged severe acute respiratory syndrome coronavirus 2 persistence, attenuated immunologic response, and viral evolution in a solid organ transplant patient. Am J Transplant. 2022 Feb;22(2):649-653. doi: 10.1111/ajt.16837. | No viral cultures |
|  | Qian Q, Fan L, Liu W, et al. Direct Evidence of Active SARS-CoV-2 Replication in the Intestine. Clin Infect Dis. 2021 Aug 2;73(3):361-366. doi: 10.1093/cid/ciaa925. | Not respiratory samples |
|  | Oordt-Speets AM, Spinardi JR, Mendoza CF, et al. Duration of SARS-CoV-2 shedding: A systematic review. J Glob Health. 2024;14:05005. doi: 10.7189/jogh.14.05005. | Systematic review |
|  | Ouoba S, Okimoto M, Nagashima S, et al. Sequential dynamics of virological and serological changes in the serum of SARS-CoV-2 infected patients. J Med Virol. 2022 Apr;94(4):1734-1737. doi: 10.1002/jmv.27518. | No viral cultures (from respiratory samples) |
|  | Owusu D, Pomeroy MA, Lewis NM, et al. Persistent SARS-CoV-2 RNA Shedding Without Evidence of Infectiousness: A Cohort Study of Individuals With COVID-19. J Infect Dis. 2021 Oct 28;224(8):1362-1371. doi: 10.1093/infdis/jiab107. | Data cannot be extracted at individual level |
|  | Raglow Z, Surie D, Chappell JD, et al. SARS-CoV-2 shedding and evolution in patients who were immunocompromised during the omicron period: a multicentre, prospective analysis. Lancet Microbe. 2024;5(3):e235-e246. doi: 10.1016/S2666-5247(23)00336-1. | Data cannot be extracted from figures |
|  | Reyes NS, Rodriguez PE, Ricarte C, Echegoyen N, Viegas M, Varese A, Ceballos A, Stryjewski ME, Echavarria M. Shedding of infectious SARS-CoV-2 in two asymptomatic children. Medicina (B Aires). 2023;83(2):185-189. | Not adults |
|  | Rhee C, Kanjilal S, Baker M, Klompas M. Duration of Severe Acute Respiratory Syndrome Coronavirus 2 (SARS-CoV-2) Infectivity: When Is It Safe to Discontinue Isolation? Clin Infect Dis. 2021 Apr 26;72(8):1467-1474. doi: 10.1093/cid/ciaa1249. | Review |
|  | Riemersma KK, Haddock LA 3rd, Wilson NA, et al. Shedding of infectious SARS-CoV-2 despite vaccination. PLoS Pathog. 2022 Sep 30;18(9):e1010876. doi: 10.1371/journal.ppat.1010876. | Not serial Cts. Aggregated data |
|  | Robinson ML, Mirza A, Gallagher N, et al. Limitations of Molecular and Antigen Test Performance for SARS-CoV-2 in Symptomatic and Asymptomatic COVID-19 Contacts. J Clin Microbiol. 2022 Jul 20;60(7):e0018722. doi: 10.1128/jcm.00187-22. | Aggregated data |
|  | Roedl K, Heidenreich S, Pfefferle S, et al. Viral Dynamics of SARS-CoV-2 in Critically Ill Allogeneic Hematopoietic Stem Cell Transplant Recipients and Immunocompetent Patients with COVID-19. Am J Respir Crit Care Med. 2021 Jan 15;203(2):242-245. doi: 10.1164/rccm.202009-3386LE. | Data cannot be extracted at individual level |
|  | Rodríguez-Grande C, Adán-Jiménez J, Catalán P, et al. Inference of Active Viral Replication in Cases with Sustained Positive Reverse Transcription-PCR Results for SARS-CoV-2. J Clin Microbiol. 2021 Jan 21;59(2):e02277-20. doi: 10.1128/JCM.02277-20. | No viral cultures |
|  | Rozenberg G, Erster O, Ghersin I, et al. Evaluation of the relationship between quantitative PCR results and cell culturing of SARS2-CoV with respect to symptoms onset and Viral load – a systematic review. medRxiv 2021.08.23.21262162; doi: <https://doi.org/10.1101/2021.08.23.21262162> | Systematic review |
|  | Sahbudak Bal Z, Ozkul A, Bilen M, Kurugol Z, Ozkinay F. The Longest Infectious Virus Shedding in a Child Infected With the G614 Strain of SARS-CoV-2. Pediatr Infect Dis J. 2021 Jul 1;40(7):e263-e265. doi: 10.1097/INF.0000000000003158. | No data on Ct |
|  | Salaouatchi MT, Mahadeb B, Clevenbergh P, et al. Efficacy of systematic coronavirus screening by PCR and viral cultures in addition to triage in limiting the spread of SARS-CoV-2 within a hemodialysis unit. J Nephrol. 2022 Jan;35(1):113-120. doi: 10.1007/s40620-021-01115-w. | Aggregated data |
|  | Salvatore PP, Lee CC, Sleweon S, et al. Transmission potential of vaccinated and unvaccinated persons infected with the SARS-CoV-2 Delta variant in a federal prison, July—August 2021. medRxiv 2021.11.12.21265796; doi: https://doi.org/10.1101/2021.11.12.21265796 | Data cannot be extracted at individual level |
|  | Salvatore PP, Lee CC, Sleweon S, McCormick DW, et al. Transmission potential of vaccinated and unvaccinated persons infected with the SARS-CoV-2 Delta variant in a federal prison, July-August 2021. Vaccine. 2023 Mar 10;41(11):1808-1818. doi: 10.1016/j.vaccine.2022.11.045. Epub 2022 Dec 13. PMID: 36572604; PMCID: PMC9744684. | Aggregated data. Also published on MedRxiv ( https://doi.org/10.1101/2021.11.12.21265796) |
|  | Santarpia JL, Rivera DN, Herrera VL, et al. Aerosol and surface contamination of SARS-CoV-2 observed in quarantine and isolation care. Sci Rep. 2020 Jul 29;10(1):12732. doi: 10.1038/s41598-020-69286-3. Erratum in: Sci Rep. 2020 Aug 12;10(1):13892. | environmental study |
|  | Santarpia JL, Herrera VL, Rivera DN, et al. The size and culturability of patient-generated SARS-CoV-2 aerosol. J Expo Sci Environ Epidemiol. 2022 Sep;32(5):706-711. | Environmental study |
|  | Santos Bravo M, Nicolás D, Berengua C, et al. Severe Acute Respiratory Syndrome Coronavirus 2 Normalized Viral Loads and Subgenomic RNA Detection as Tools for Improving Clinical Decision Making and Work Reincorporation. J Infect Dis. 2021 Oct 28;224(8):1325-1332. doi: 10.1093/infdis/jiab394. PMID: 34329473; PMCID: PMC8436374. | No viral cultures |
|  | Seike I, Baba H, Okamoto M, et al. Validation of the United States isolation termination criteria using virus culture results of the omicron variant in Japan. J Infect Chemother. 2025;31(6):102714. doi: 10.1016/j.jiac.2025.102714. | Aggregated data |
|  | Sejdic A, Frische A, Jørgensen CS, et al. High titers of neutralizing SARS-CoV-2 antibodies six months after symptom onset are associated with increased severity in COVID-19 hospitalized patients. Virol J. 2023 Jan 25;20(1):14. doi: 10.1186/s12985-023-01974-8. | Aggregated data |
|  | Selby LM, Hewlett A, Wood MG, Starlin R. Evaluation of cycle threshold to assist with safe return to work for healthcare workers with coronavirus disease 2019 (COVID-19). Infect Control Hosp Epidemiol. 2023 Apr;44(4):681-682. doi: 10.1017/ice.2022.252. | No viral cultures |
|  | Singanayagam A, Patel M, Charlett A, et al. Duration of infectiousness and correlation with RT-PCR cycle threshold values in cases of COVID-19, England, January to May 2020. Euro Surveill. 2020 Aug;25(32):2001483. doi: 10.2807/1560-7917.ES.2020.25.32.2001483. Erratum in: Euro Surveill. 2021 Feb;26(7) | Aggregated data |
|  | Sohn Y, Jeong SJ, Chung WS, et al. Assessing Viral Shedding and Infectivity of Asymptomatic or Mildly Symptomatic Patients with COVID-19 in a Later Phase. J Clin Med. 2020 Sep 10;9(9):2924. doi: 10.3390/jcm9092924. | Aggregated data |
|  | Srichatrapimuk S, Chookajorn T, Kochakarn T, et al. SARS-CoV-2 RT-PCR positivity of individuals subsequent to completing quarantine upon entry into a country during a transmission-free period. Travel Med Infect Dis. 2022 Mar-Apr;46:102271. doi: 10.1016/j.tmaid.2022.102271. | The date of viral culture is not specified |
|  | Surie D, Huang JY, Brown AC, et al. Infectious Period of Severe Acute Respiratory Syndrome Coronavirus 2 in 17 Nursing Home Residents-Arkansas, June-August 2020. Open Forum Infect Dis. 2021 Jan 30;8(3): ofab048. doi: 10.1093/ofid/ofab048. | Data cannot be extracted at individual level |
|  | Suzuki M, Imai T, Sakurai A, et al. Virological and genomic analysis of SARS-CoV-2 from a favipiravir clinical trial cohort. J Infect Chemother. 2021 Sep;27(9):1350-1356. doi: 10.1016/j.jiac.2021.06.010. | Aggregated data |
|  | Tallmadge RL, Laverack M, Cronk B, et al. Viral load and infectivity of SARS-CoV-2 in paired respiratory and oral specimens from symptomatic, asymptomatic or post-symptomatic individuals. medRxiv 2021.11.13.21266305; doi: https://doi.org/10.1101/2021.11.13.21266305 | No serial Cts |
|  | Taylor J, Carter RJ, Lehnertz N, et al. Serial Testing for SARS-CoV-2 and Virus Whole Genome Sequencing Inform Infection Risk at Two Skilled Nursing Facilities with COVID-19 Outbreaks - Minnesota, April-June 2020. MMWR Morb Mortal Wkly Rep. 2020 Sep 18;69(37):1288-1295. doi: 10.15585/mmwr.mm6937a3. | No viral cultures |
|  | To KK, Tsang OT, Leung WS, et al. Temporal profiles of viral load in posterior oropharyngeal saliva samples and serum antibody responses during infection by SARS-CoV-2: an observational cohort study. Lancet Infect Dis. 2020 May;20(5):565-574. doi: 10.1016/S1473-3099(20)30196-1. | No data on viral cultures |
|  | Tobolowsky FA, Waltenburg MA, Moritz ED, et al. Longitudinal serologic and viral testing post–SARS-CoV-2 infection and post-receipt of mRNA COVID-19 vaccine in a nursing home cohort—Georgia, October 2020‒April 2021. medRxiv 2021.12.28.21268458; doi: https://doi.org/10.1101/2021.12.28.21268458 | Published in PLoS (preprint of doi: 10.1371/journal.pone.0275718) |
|  | Tu YP, Green C, Hao L, et al. COVID-19 Antigen Results Correlate with the Quantity of Replication-Competent SARS-CoV-2 in a Cross-Sectional Study of Ambulatory Adults during the Delta Wave. Microbiol Spectr. 2023;11(3):e0006423. doi: 10.1128/spectrum.00064-23. | Data cannot be extracted at individual level |
|  | Van Cleemput J, van Snippenberg W, Lambrechts L,et al. Organ-specific genome diversity of replication-competent SARS-CoV-2. Nat Commun. 2021 Nov 16;12(1):6612. doi: 10.1038/s41467-021-26884-7. Erratum in: Nat Commun. 2022 Oct 21;13(1):6247. | Post-mortem study |
|  | van Kampen JJA, van de Vijver DAMC, Fraaij PLA, et al. Duration and key determinants of infectious virus shedding in hospitalized patients with coronavirus disease-2019 (COVID-19). Nat Commun. 2021 Jan 11;12(1):267, | Data cannot be extracted at individual level |
|  | Viana LA, Cristelli MP, Ficher KN, et al. Kidney Transplantation in Patients With SARS-CoV-2 Infection: A Case Series Report. Transplantation. 2021 Jan 1;105(1):e1-e3. doi: 10.1097/TP.0000000000003521. | No data on serial Cts |
|  | Virata MD, Villanueva M, Shenoi S, et al. SARS-CoV-2 Viral Dynamics For Symptomatic People Living with HIV Requiring Hospitalization For COVID-19, Open Forum Infectious Diseases, Volume 7, Issue Supplement_1, October 2020, Page S338, https://doi.org/10.1093/ofid/ofaa439.737 | No viral cultures (meeting abstract) |
|  | Walsh KA, Spillane S, Comber L,et al. The duration of infectiousness of individuals infected with SARS-CoV-2. J Infect. 2020 Dec;81(6):847-856. doi: 10.1016/j.jinf.2020.10.009. | Review |
|  | Wang W, Xu Y, Gao R, et al. Detection of SARS-CoV-2 in Different Types of Clinical Specimens. JAMA. 2020 May 12;323(18):1843-1844. doi: 10.1001/jama.2020.3786. | Aggregated data |
|  | Walsh KA, Spillane S, Comber L, et al. The duration of infectiousness of individuals infected with SARS-CoV-2. J Infect. 2020 Dec;81(6):847-856. doi: 10.1016/j.jinf.2020.10.009. | Rapid review |
|  | Wölfel R, Corman VM, Guggemos W, et al. Virological assessment of hospitalized patients with COVID-2019. Nature. 2020 May;581(7809):465-469. doi: 10.1038/s41586-020-2196-x. Erratum in: Nature. 2020 Dec;588(7839):E35.. | Data cannot be extracted at individual level |
|  | Wu Y, Guo Z, Yuan J, et al. Duration of viable virus shedding and polymerase chain reaction positivity of the SARS-CoV-2 Omicron variant in the upper respiratory tract: a systematic review and meta-analysis. Int J Infect Dis. 2023;129:228-235. doi: 10.1016/j.ijid.2023.02.011. | Systematic review |
|  | Xiao F, Sun J, Xu Y, et al. Infectious SARS-CoV-2 in Feces of Patient with Severe COVID-19. Emerg Infect Dis. 2020 Aug;26(8):1920-1922. doi: 10.3201/eid2608.200681 | not respiratory samples |
|  | Xiao F, Tang M, Zheng X, Liu Y, Li X, Shan H. Evidence for Gastrointestinal Infection of SARS-CoV-2. Gastroenterology. 2020 May;158(6):1831-1833.e3. doi: 10.1053/j.gastro.2020.02.055. | not respiratory samples |
|  | Yao H, Lu X, Chen Q, et al. Patient-derived SARS-CoV-2 mutations impact viral replication dynamics and infectivity in vitro and with clinical implications in vivo. Cell Discov. 2020 Oct 29;6(1):76. doi: 10.1038/s41421-020-00226-1. | no data on Cts of respiratory samples |
|  | Young BE, Ong SWX, Ng LFP, et al. Viral Dynamics and Immune Correlates of Coronavirus Disease 2019 (COVID-19) Severity. Clin Infect Dis. 2021 Nov 2;73(9):e2932-e2942. doi: 10.1093/cid/ciaa1280. | Data cannot be extracted at an individual level |
|  | Yuan Y, Wang H, Zhao J, et al. Severe Acute Respiratory Syndrome Coronavirus 2 Viral RNA Load Status and Antibody Distribution Among Patients and Asymptomatic Carriers in Central China. Front Cell Infect Microbiol. 2021 Mar 19;11:559447. doi: 10.3389/fcimb.2021.559447. | No viral cultures |
|  | Zacharioudakis IM, Zervou FN, Prasad PJ, et al Association of SARS-CoV-2 genomic load trends with clinical status in COVID-19: A retrospective analysis from an academic hospital center in New York City. PLoS One. 2020 Nov 17;15(11):e0242399. doi: 10.1371/journal.pone.0242399. | No viral cultures |
|  | Zhang Y, Chen C, Zhu S, et al. Isolation of 2019-nCoV from a Stool Specimen of a Laboratory-Confirmed Case of the Coronavirus Disease 2019 (COVID-19). China CDC Wkly. 2020 Feb 21;2(8):123-124. | Data cannot be extracted at an individual level |
|  | Zhou J, Otter JA, Price JR, et al. Investigating Severe Acute Respiratory Syndrome Coronavirus 2 (SARS-CoV-2) Surface and Air Contamination in an Acute Healthcare Setting During the Peak of the Coronavirus Disease 2019 (COVID-19) Pandemic in London. Clin Infect Dis. 2021 Oct 5;73(7):e1870-e1877. doi: 10.1093/cid/ciaa905. | Not respiratory samples; environmental study |
