# Supplementary material for: Serial cycle threshold to assess the infectious potential of SARS-CoV-2: A systematic review
Source: Epidemiol Infect. 2026 May 6;154:e89. doi: 10.1017/S0950268826101484 (PMC13366375; doi:10.1017/S0950268826101484)
Supplement: Rosca et al. supplementary material [file S0950268826101484sup001.zip › Appendix 3. List of included studies.docx]

**Supplementary Material S - List of included studies**

1. Alshukairi AN, Tolah AM, Dada A, et al. Test-based de-isolation in COVID-19 immunocompromised patients: Cycle threshold value versus SARS-CoV-2 viral culture. Int J Infect Dis. 2021; 108:112-115. doi: 10.1016/j.ijid.2021.05.027.
2. Avanzato VA, Matson MJ, Seifert SN, et al. Case Study: Prolonged Infectious SARS-CoV-2 Shedding from an Asymptomatic Immunocompromised Individual with Cancer. Cell. 2020; 183(7):1901-1912.e9. doi: 10.1016/j.cell.2020.10.049.
3. Aydillo T, Gonzalez-Reiche AS, Aslam S, et al. Shedding of Viable SARS-CoV-2 after Immunosuppressive Therapy for Cancer. N Engl J Med. 2020; 383(26):2586-2588. doi: 10.1056/NEJMc2031670.
4. Baang JH, Smith C, Mirabelli C, et al. Prolonged Severe Acute Respiratory Syndrome Coronavirus 2 Replication in an Immunocompromised Patient. J Infect Dis. 2021; 223(1):23-27. doi: 10.1093/infdis/jiaa666.
5. Basheer M, Saad E, Laskar O, et al. Clearance of the SARS-CoV-2 Virus in an Immunocompromised Patient Mediated by Convalescent Plasma without B-Cell Recovery. Int J Mol Sci. 2021; 22(16):8902. doi: 10.3390/ijms22168902.
6. Choi B, Choudhary MC, Regan J, et al. Persistence and Evolution of SARS-CoV-2 in an Immunocompromised Host. N Engl J Med. 2020 Dec 3;383(23):2291-2293. doi: 10.1056/NEJMc2031364.
7. Decker A, Welzel M, Laubner K, et al. Prolonged SARS-CoV-2 shedding and mild course of COVID-19 in a patient after recent heart transplantation. Am J Transplant. 2020; 20(11):3239-3245. doi: 10.1111/ajt.16133.
8. Garcia-Knight M, Anglin K, Tassetto M, et al. Infectious viral shedding of SARS-CoV-2 Delta following vaccination: A longitudinal cohort study. PLoS Pathog. 2022 Sep 12;18(9):e1010802. doi: 10.1371/journal.ppat.1010802.
9. Gniazdowski V, Paul Morris C, Wohl S, et al. Repeated Coronavirus Disease 2019 Molecular Testing: Correlation of Severe Acute Respiratory Syndrome Coronavirus 2 Culture With Molecular Assays and Cycle Thresholds. Clin Infect Dis. 2021; 73(4):e860-e869. doi: 10.1093/cid/ciaa1616.
10. Guetl K, Moazedi-Fuerst F, Rosskopf K, et al. SARS-CoV-2 positive virus culture 7 weeks after onset of COVID-19 in an immunocompromised patient suffering from X chromosome-linked agammaglobulinemia. J Infect. 2021;82(3):414-451. doi: 10.1016/j.jinf.2020.10.025.
11. Han A, Rodriguez TE, Beck ET, et al. Persistent SARS-CoV-2 infectivity greater than 50 days in a case series of allogeneic peripheral blood stem cell transplant recipients. Curr Probl Cancer Case Rep. 2021; 3:100057. doi: 10.1016/j.cpccr.2021.100057.
12. Jung J, Kang SW, Lee S, et al. Risk of transmission of COVID-19 from healthcare workers returning to work after a 5-day isolation, and kinetics of shedding of viable SARS-CoV-2 variant B.1.1.529 (Omicron). J Hosp Infect. 2023; 131:228-233. doi: 10.1016/j.jhin.2022.11.012.
13. Kang SW, Park H, Kim JY, et al. Comparison of the clinical and virological characteristics of SARS-CoV-2 Omicron BA.1/BA.2 and omicron BA.5 variants: A prospective cohort study. J Infect. 2023 May;86(5):e148-e151. doi: 10.1016/j.jinf.2023.01.015.
14. Ke R, Martinez PP, Smith RL, et al. Daily sampling of early SARS-CoV-2 infection reveals substantial heterogeneity in infectiousness. medRxiv [Preprint]. 2021 Jul 12:2021.07.12.21260208. doi: 10.1101/2021.07.12.21260208. Update in: Nat Microbiol. 2022 May;7(5):640-652.
15. Kim JY, Bae JY, Bae S, et al. Diagnostic usefulness of subgenomic RNA detection of viable SARS-CoV-2 in patients with COVID-19. Clin Microbiol Infect. 2022; 28(1):101-106. doi: 10.1016/j.cmi.2021.08.009.
16. Kim DY, Lin MY, Jennings C, et al. Duration of Replication-Competent Severe Acute Respiratory Syndrome Coronavirus 2 (SARS-CoV-2) Shedding Among Patients With Severe or Critical Coronavirus Disease 2019 (COVID-19). Clin Infect Dis. 2023 Feb 8;76(3):e416-e425. doi: 10.1093/cid/ciac405.
17. Kujawski SA, Wong KK, Collins JP, et al. (COVID-19 Investigation Team). Clinical and virologic characteristics of the first 12 patients with coronavirus disease 2019 (COVID-19) in the United States. Nat Med. 2020;26(6):861-868. doi: 10.1038/s41591-020-0877-5.
18. Laferl H, Kelani H, Seitz T, et al. An approach to lifting self-isolation for health care workers with prolonged shedding of SARS-CoV-2 RNA. Infection. 2021; 49(1):95-101. doi: 10.1007/s15010-020-01530-4.
19. Lang C, Jaksch P, Hoda MA, et al. Lung transplantation for COVID-19-associated acute respiratory distress syndrome in a PCR-positive patient. Lancet Respir Med. 2020; 8(10):1057-1060. doi: 10.1016/S2213-2600(20)30361-1.
20. Leitão IC, Calil PT, Galliez RM, et al. Prolonged SARS-CoV-2 Positivity in Immunocompetent Patients: Virus Isolation, Genomic Integrity, and Transmission Risk. Microbiol Spectr. 2021; 9(3):e0085521. doi: 10.1128/Spectrum.00855-21.
21. Leung WF, Chorlton S, Tyson Jet al. COVID-19 in an immunocompromised host: persistent shedding of viable SARS-CoV-2 and emergence of multiple mutations: a case report. Int J Infect Dis. 2022; 114:178-182. doi: 10.1016/j.ijid.2021.10.045.
22. Lin YC, Malott RJ, Ward L, et al. Detection and quantification of infectious severe acute respiratory coronavirus-2 in diverse clinical and environmental samples. Sci Rep. 2022; 12(1):5418. doi: 10.1038/s41598-022-09218-5.
23. Lu J, Peng J, Xiong Q, et al. Clinical, immunological and virological characterization of COVID-19 patients that test re-positive for SARS-CoV-2 by RT-PCR. EBioMedicine. 2020; 59:102960. doi: 10.1016/j.ebiom.2020.102960.
24. Luna-Muschi A, Noguera SV, Borges IC, et al. Characterization of Severe Acute Respiratory Syndrome Coronavirus 2 Omicron Variant Shedding and Predictors of Viral Culture Positivity on Vaccinated Healthcare Workers With Mild Coronavirus Disease 2019. J Infect Dis. 2022 Nov 11;226(10):1726-1730. doi: 10.1093/infdis/jiac391.
25. Mancon A, Rizzo A, Mileto D, et al. Viro-immunological evaluation in an immunocompromised patient with long-lasting SARS-CoV-2 infection. Emerg Microbes Infect. 2022; 11(1):786-789. doi: 10.1080/22221751.2022.2045877.
26. McCormick DW, Hagan LM, Salvatore PP, et al. SARS-CoV-2 viral shedding in vaccinated and unvaccinated persons: A case series. Vaccine. 2023 Mar 10;41(11):1769-1773. doi: 10.1016/j.vaccine.2022.11.030.
27. Mendes-Correa MC, Ghilardi F, Chiarastelli Salomão M, et al. SARS-CoV-2 shedding, infectivity and evolution in an immunocompromised adult patient. medRxiv 2021.06.11.21257717; doi: <https://doi.org/10.1101/2021.06.11.21257717>
28. Mileto D, Foschi A, Mancon A, et al. A case of extremely prolonged viral shedding: Could cell cultures be a diagnostic tool to drive COVID-19 patient discharge? Int J Infect Dis. 2021; 104:631-633. doi: 10.1016/j.ijid.2020.11.161
29. Monrad I, Sahlertz SR, Nielsen SSF, et al. Persistent Severe Acute Respiratory Syndrome Coronavirus 2 Infection in Immunocompromised Host Displaying Treatment Induced Viral Evolution. Open Forum Infect Dis. 2021; 8(7):ofab295. doi: 10.1093/ofid/ofab295.
30. Murata T, Sakurai A, Suzuki M, et al. Shedding of Viable Virus in Asymptomatic SARS-CoV-2 Carriers. mSphere. 2021; 6(3):e00019-21. doi: 10.1128/mSphere.00019-21.
31. Nissen K, Hagbom M, Krambrich J, et al. Presymptomatic viral shedding and infective ability of SARS-CoV-2; a case report. Heliyon. 2021; 7(2):e06328. doi: 10.1016/j.heliyon.2021.e06328.
32. Niyonkuru M, Pedersen RM, Assing K, et al. Prolonged viral shedding of SARS-CoV-2 in two immunocompromised patients, a case report. BMC Infect Dis. 2021; 21(1):743. doi: 10.1186/s12879-021-06429-5.
33. Nomura T, Kitagawa H, Omori K, et al. Duration of infectious virus shedding in patients with severe coronavirus disease 2019 who required mechanical ventilation. J Infect Chemother. 2022; 28(1):19-23. doi: 10.1016/j.jiac.2021.09.006.
34. Pedro N, Silva CN, Magalhães AC, et al. Dynamics of a Dual SARS-CoV-2 Lineage Co-Infection on a Prolonged Viral Shedding COVID-19 Case: Insights into Clinical Severity and Disease Duration. Microorganisms. 2021; 9(2):300. doi: 10.3390/microorganisms9020300.
35. Pérez-Lago L, Aldámiz-Echevarría T, García-Martínez R, et al. Different Within-Host Viral Evolution Dynamics in Severely Immunosuppressed Cases with Persistent SARS-CoV-2. Biomedicines. 2021; 9(7):808. doi: 10.3390/biomedicines9070808.
36. Pickering S, Batra R, Merrick B, et al. Comparative performance of SARS-CoV-2 lateral flow antigen tests and association with detection of infectious virus in clinical specimens: a single-centre laboratory evaluation study. Lancet Microbe. 2021; 2(9):e461-e471. doi: 10.1016/S2666-5247(21)00143-9. Erratum in: Lancet Microbe. 2021 Sep;2(9):e426.
37. Rajakumar I, Isaac DL, Fine NM, et al. Extensive environmental contamination and prolonged severe acute respiratory coronavirus-2 (SARS CoV-2) viability in immunosuppressed recent heart transplant recipients with clinical and virologic benefit with remdesivir. Infect Control Hosp Epidemiol. 2022; 43(6):817-819. doi: 10.1017/ice.2021.89.
38. Sepulcri C, Dentone C, Mikulska M, et al. The Longest Persistence of Viable SARS-CoV-2 With Recurrence of Viremia and Relapsing Symptomatic COVID-19 in an Immunocompromised Patient-A Case Study. Open Forum Infect Dis. 2021; 8(11):ofab217. doi: 10.1093/ofid/ofab217.
39. Siedner MJ, Boucau J, Gilbert RF, et al. Duration of viral shedding and culture positivity with postvaccination SARS-CoV-2 delta variant infections. JCI Insight. 2022; 7(2):e155483. doi: 10.1172/jci.insight.155483.
40. Singh AK, Stellrecht KA, Arunachalam T, et al. Lack of active SARS-CoV-2 virus in a subset of PCR-positive COVID-19 congregate care patients. J Clin Virol. 2021; 141:104879. doi: 10.1016/j.jcv.2021.104879.
41. Spinicci M, Mazzoni A, Coppi M, et al. Long-term SARS-CoV-2 Asymptomatic Carriage in an Immunocompromised Host: Clinical, Immunological, and Virological Implications. J Clin Immunol. 2022 Oct;42(7):1371-1378. doi: 10.1007/s10875-022-01313-6.
42. Sung A, Bailey AL, Stewart HB, et al. Isolation of SARS-CoV-2 in Viral Cell Culture in Immunocompromised Patients With Persistently Positive RT-PCR Results. Front Cell Infect Microbiol. 2022; 12:804175. doi: 10.3389/fcimb.2022.804175.
43. Tarhini H, Recoing A, Bridier-Nahmias A, et al. Long-Term Severe Acute Respiratory Syndrome Coronavirus 2 (SARS-CoV-2) Infectiousness Among Three Immunocompromised Patients: From Prolonged Viral Shedding to SARS-CoV-2 Superinfection. J Infect Dis. 2021; 223(9):1522-1527. doi: 10.1093/infdis/jiab075.
44. Thornton CS, Huntley K, Berenger BM, et al. Prolonged SARS-CoV-2 infection following rituximab treatment: clinical course and response to therapeutic interventions correlated with quantitative viral cultures and cycle threshold values. Antimicrob Resist Infect Control. 2022; 11(1):28. doi: 10.1186/s13756-022-01067-1.
45. Tobolowsky FA, Waltenburg MA, Moritz ED, et al. Longitudinal serologic and viral testing post-SARS-CoV-2 infection and post-receipt of mRNA COVID-19 vaccine in a nursing home cohort-Georgia, October 2020‒April 2021. PLoS One. 2022 Oct 27;17(10):e0275718. doi: 10.1371/journal.pone.0275718.
46. Truong TT, Ryutov A, Pandey U, et al. Increased viral variants in children and young adults with impaired humoral immunity and persistent SARS-CoV-2 infection: A consecutive case series. EBioMedicine. 2021; 67:103355. doi: 10.1016/j.ebiom.2021.103355.
47. Weigang S, Fuchs J, Zimmer G, et al. Within-host evolution of SARS-CoV-2 in an immunosuppressed COVID-19 patient as a source of immune escape variants. Nat Commun. 2021; 12(1):6405. doi: 10.1038/s41467-021-26602-3
48. Williamson MK, Hamilton F, Hutchings S, et al. Chronic SARS-CoV-2 infection and viral evolution in a hypogammaglobulinaemic individual. medRxiv 2021.05.31.21257591; doi: <https://doi.org/10.1101/2021.05.31.21257591>
49. Zahn T, Mhedhbi I, Hein S, et al. Persistence of infectious SARS-CoV-2 particles for up to 37 days in patients with mild COVID-19. Allergy. 2022; 77(7):2053-2066. doi: 10.1111/all.15138.
50. Zupin L, Fontana F, Clemente L, et al. Persistent viral infectivity after 27 days from COVID-19 symptoms onset. J Clin Pathol. 2022; 75(3):211-214. doi: 10.1136/jclinpath-2021-207394.
