# Supplementary material for: Serial cycle threshold to assess the infectious potential of SARS-CoV-2: A systematic review
Source: Epidemiol Infect. 2026 May 6;154:e89. doi: 10.1017/S0950268826101484 (PMC13366375; doi:10.1017/S0950268826101484)
Supplement: Rosca et al. supplementary material [file S0950268826101484sup001.zip › WebTable 1. Symptoms, veracity of symptom, medical history.docx]

**WebTable 1. Symptoms, veracity of symptom and signs, and medical history**

| **Study** | **Symptoms** | **Veracity of symptom & sign checking** | **Medical history / Other Clinical data** |
| --- | --- | --- | --- |
| Alshukairi 2021 a | Symptomatic | Charts were reviewed for demographics, comorbidities, clinical course, outcome, and immunosuppressive medication | P1: Cardiac transplant, epilepsy. P2: Renal transplant, diabetes mellitus, hypertension, coronary artery disease. P3: Renal transplant, hypertension, benign prostatic hyperplasia. P4: Lymphoma. P5: Renal transplant, diabetes mellitus. P6: Renal transplant, anti-phospholipid syndrome, hypothyroidism. P7: Hepatocellular cancer, diabetes mellitus, hypertension, ischemic heart disease, chronic liver disease. P8: Diabetes mellitus, hypertension, hypopituitarism. P9: Von Willebrand disease. P10: Diabetes mellitus, hypertension, coronary artery disease. P11: Diabetes mellitus, hypertension. P12: Asthma, hypertension. P13: Hypothyroidism |
| Avanzato 2021 | Asymptomatic | N/R | Chronic lymphocytic leukemia; acquired hypogammaglobulinemia. During the course of the study, the patient was transfused with intravenous immunoglobulin (IVIG, 25 g) on D35 and 65, and convalescent plasma against SARS-CoV-2 on D71 and D82 |
| Aydillo 2020 | Symptomatic (n=11) | The patients’ demographic characteristics, medical history, and clinical course of Covid-19 were abstracted from medical records. | P1: Hematopoietic stem-cell transplants. P2: Hematopoietic stem-cell transplants. P3: Lymphoma. CAR-T recipient. P4: Hematopoietic stem-cell transplants. P5: Hematopoietic stem-cell transplants. P6: Hematopoietic stem-cell transplants. P7: Hematopoietic stem-cell transplants. P8: Lymphoma. P9: Hematopoietic stem-cell transplants. P10: Hematopoietic stem-cell transplants. P11: Lymphoma, CAR-T recipient |
| Baang 2021 | Symptomatic. | N/R | Refractory mantle cell lymphoma (immunohistochemistry positive for CD20, CD5, BCL2, cyclin D1, and SOX11; lambda-restricted, CD5-positive B-cell population by flow cytometry). Chemotherapy-associated neutropenia and severe thrombocytopenia. D0 - immunochemotherapy for lymphoma was ongoing and included a CD20 bispecific antibody and a second B-cell directed antibody in combination with cyclophosphamide, doxorubicin, and prednisone. |
| Basheer 2021 | Asymptomatic on D1. Symptomatic | N/R | Treatment with Rituximab. First hospitalization (D25-D41): convalescent plasma. Second hospitalization (D61-D70): Remdesivir (10 days), convalescent plasma, IVIG, ivermectin. |
| Decker 2020 | Mild symptoms (D1-D20). D1- fever (39.9°C), tachycardia (105 bpm), and a sore throat. | N/R | Heart transplant. Anaemia, leukopenia. Immunosuppression regimen: cyclosporine A (target range 135 ± 30 ng/mL), mycophenolate mofetil 500 mg b.i.d., and prednisone 10 mg q.d. He received cotrimoxazole and due to cytomegalovirus (CMV) high-risk constellation, ganciclovir was administered for 4 months after transplantation and was then switched to valganciclovir prophylaxis. Hydroxychloroquine (loading dose 400 mg b.i.d. followed by 200 mg b.i.d.) from D7 to D14. |
| Gniazdowski 2021 | Symptomatic | Clinical data were extracted by manual chart reviews. | 24/26 patients with viral cultures had ≥ 2 chronic conditions; 5/29 patients with viral cultures necessitated ICU; 13/26 patients were hospitalized; 8/26 patients were ambulatory . Immunodeficient (chemotherapy, transplant recipient) - 13/57 |
| Guetl 2021 | Symptomatic. Severe COVID-19 (ICU starting D29). | N/R | X chromosome-linked agammaglobulinemia (XLA). Starting with hospital admission (D16), the patient received antibiotic treatment with amoxicillin /clavulanic acid and azithromycin, was switched to piperacillin/tazobactam, followed by moxifloxacin and meropenem. Interleukin-6 (IL-6) receptor blockade by tocilizumab and convalescent plasma were administered on D30. Convalescent plasma transfusion was repeated on D41. |
| Han 2021 | Symptomatic. | N/R | X chromosome-linked agammaglobulinemia (XLA). The patient was treated with the CALGB 10,403 regimen, followed by vincristine, daunorubicin, PEG asparaginase, and intrathecal methotrexate plus cytarabine. PBSCT with a conditioning regimen of busulfan, fludarabine, and cyclophosphamide. Graft versus host disease (GVHD) prophylaxis consisted of post-transplant cyclophosphamide, tacrolimus and mycophenolate. Mycophenolate was initiated on day 5 post-transplantation and continued for 30 days. He was treated with 24 days of ganciclovir and remdesivir (D159D19; D54-D63) |
| Jung 2023 | Symptomatic: 20  Asymptomatic: 12 | N/R | Vaccination status: 2-dose 4 (12%), 3-dose 28 (88%). |
| Ke 2021 | Symptomatic- 55 cases. Asymptomatic - 5 cases | Self-reported symptoms | COPD: 1 (1.7%). Asthma 2 (3.3%). Cancer 2 (3.3%). Immunodeficiency 1 (1.7%). Anaemia 1 (1.7%) |
| Kim 2022 | Initial symptom: Fever 18; Chills or rigors; Myalgia 5; Headache 2; Sore throat 2; Nausea or vomiting 3; Diarrhoea 0; Fatigue 3; Congestion or runny nose 1; Cough 9; Shortness of breathing 3; Difficulty of breathing 4; New olfactory disorder 2; New taste disorder 2. Hospital course: Pneumonia 16; Supplemental oxygen therapy 11; Mechanical ventilation 4; Extracorporeal membrane oxygenation 1. | N/R | P1: Diabetes, hypertension. P2: Diabetes, hypertension, COPD. P3: None. P4: None. P5: Acute myeloid leukaemia. P6: Acute lymphoblastic leukaemia. P7: None. P8: Hypertension. P9: None. P10: None. P11: None. P12: Bipolar disorder. P13: Diffuse large B cell lymphoma. P14: Hypertension. P15: Liver cirrhosis. P16: Diabetes, brain abscess. P17: Cholangiocarcinoma, diabetes. P18: Thoracic aortic aneurysm. P19: None. P20: Hypertension |
| Kujavski 2020 | Symptomatic: fever, cough, fatigue, dyspnoea, sore throat, headache, rhinorrhoea, chills, diarrhoea, nausea | Patients were interviewed by public health officials about demographics, exposures, travel history and symptoms, including signs or symptoms before presentation. For all patients, available medical records were reviewed. For hospitalized patients, clinicians systematically abstracted data from the medical record. | P6 - Hypertriglyceridemia; P7 - Hypertension, hyperlipidaemia, pacemaker for bradycardia; P8 - Tobacco use, hypertension, coronary artery disease, COPD, history of lung cancer status post partial lobectomy; P9 - Type 2 diabetes mellitus, fatty liver; P10 - None; P11 - None; P12 - None. |
| Laferl 2020 | 13 symptomatic; 2 asymptomatic. Median duration of symptoms—days 10 (range 0–38) | N/R | Co-morbidities: 4/15 (two persons with treated arterial hypertension, two with substituted hypothyroidism and one with well-controlled bronchial asthma) |
| Lang 2020 | Fever and cough (starting D0). Respiratory symptoms. Hypoxia | N/R | Mild psoriatic arthritis, which did not require any systemic treatment. Idiopathic CD4 lymphocytopenia without any clinical relevance. Convalescent plasma (D32), lung transplant (D58), daily immunoadsorption therapy (initiated on D52). After transplant - Standard triple immunosuppression with tacrolimus, mycophenolate mofetil, and steroids. Six additional treatment cycles of immunoadsorption were done and antithymocyte globulin was administered. |
| Leitão 2021 | Mild symptoms | N/R | 47.06% had no comorbidities. Arterial hypertension was the most prevalent comorbidity (23.53%), while no immunosuppressive disease/condition was reported |
| Leung 2022 | D1 - chills, sore throat and myalgia. Her initial mild symptoms resolved within 2 weeks. D33 - fever (38.7°C); progressive dyspnoea. Oxygen saturation - 70–74% on room air. D65 - acute deterioration. | N/R | Diagnosed with B-cell ALL in 2014. Allogeneic stem cell transplant in 2017 for the first relapse. Received blinatumomab for her second relapse in February 2020. Third relapse in September 2020. She received weekly doses of palliative inotuzumab in October 2020. 5 days after her third inotuzumab dose - D1 of the symptoms. D30 - fourth dose of inotuzumab. Dexamethasone starting D33. Piperacillin-tazobactam D33-D35. Trimethoprim-sulfamethoxazole (D42-D63). Voriconazole (D43–58, restarted on D65). Remdesivir (D 91–98). |
| Lin 2022 | 26 symptomatic, 1 pre-symptomatic. | A comprehensive assessment tool covering symptoms and signs (new or worsening) associated with COVID-19 was used for all inpatient cases, including core respiratory and gastrointestinal symptoms and signs and a COVID-19 expanded list including headache, muscle/joint pain, fatigue/extreme exhaustion, nausea/sudden loss of appetite, conjunctivitis/ red eye/conjunctival oedema, loss of/change to sense of smell or taste and any additional COVID-19 symptoms at the clinician’s discretion (e.g. cutaneous manifestations such as “COVID toes”). This assessment was supplemented by review of medical inpatient records and assessment by an innovative electronic clinical decision support COVID-19 symptom monitoring tool administered up to three-times daily. Serial follow up was used as required for outpatients, to improve sensitivity and reduce anchoring, recall, selection and inadequate follow up bias | P1: congestive heart failure, atrial fibrillation, anaemia, on anticoagulants. P2: DM2, Hypothyroid, Ethanol abuse, Restless leg syndrome, cerebrovascular accident, COPD, Methicillin-resistant Staphylococcus aureus (MRSA) and vancomycin-resistant enterococci +, hepatocellular carcinoma, hepatosplenomegaly, Cirrhosis, ex smoker, morbid obesity, Edema / Chronic Cellulitis; Chronic right leg wound. P3: HTN, depression, new onset DM2, Old frostbit to bilateral hands, hyponatremia/hypokalemia, delusional disorder, leg and abdominal wall cellulitis. P4: Renal Failure (Hemodialysis), OA, RA, Diabetes type 2 (lost all toes)- insulin, CAD, Angina, HTN, pacemaker, COPD/Emphysema, Seizure disorder, B cell lymphoma, memory and cognitive disorders. P5: Diagnosed Feb 2020 with classic Hairy Cell Leukemia CD19+, CD20+, CD5-/+, CD10-, CD43-, CD11c+, CD103-/+, BRAF V600E+. significant anemia of 74g/l and thrombocytopenia of 79G/L but no monocytopenia, w/o infections and mild fatigue. BM infiltration around 80%. Type 2 diabetes, High blood pressure , Dyslipidemia. P6: None. P7: Morbid obesity. Hypertension. Depression. Dyslipidemia. Gout. Previous stage IIB endometrial cancer post total abdominal hysterectomy and RT x. P8: Esophageal cancer: T3 N0 M0 squamous cell carcinoma of the esophagus. GERD: Following esophagectomy. Remote tuberculosis 40 years ago. Pulmonary tuberculosis in March 2019: Isolate was sensitive to all first line medications; completed treatment in October of 2019. Fibrotic interstitial lung disease. P9: Chronic Kidney Disease. Coronary Artery Disease. MR (mild). Paroxysmal a. fib. OSA. MDS. HTN. OA -Resulting in DDD. PUD. Gout. Recurrent UTIs. Vaginal Prolapse. Hiatal Hernia. Constipation. Bare metal stent to right coronary artery in 2017, Right total knee replacement in 2011, cholecystectomy and appendectomy in 2011, Urethral bulking in 2014. P10: Alcohol use disorder. Iron deficiency anemia. Hepatitis C. Major depressive disorder. P11: Multiple admissions to hospital for a community-acquired pneumonia (COVID-19 and viral panel negative). Multiple admissions with alcohol withdrawal, seizures. Remote history of congestive heart failure: 2D echocardiogram (2019), demonstrated normal left and right ventricular size and function; however, there was mild biatrial enlargement with possible diastolic dysfunction. Unconfirmed asthma (on Symbicort). Polysubstance abuse. Hepatitis C (negative RNA January 2019). P12: EtOH abuse, steatohepatitis, homeless, smoker, chronic issues of mood disorder, not fully characterized, diabetes mellitus type 2, diet managed, Methamphetamine and benzodiazepine abuse. Ectopic pregnancy x2 in March 2019, Cholecystectomy in the past. P13: Allergies, post nasal drip, chronic sinusitis, diabetes, gout, dyslipidemia, hypothyroidism. P14: Focal epilepsy of unknown etiology, left hemisphere. Depression. P15: None. P16: None. P17: Asthma. P18: None. P19: None. P20: Stage 4 breast cancer, prostate cancer and previous prostectomy, osteoarthritis, dyslipidemia. P21: Diabetes Type 2, diet controlled, overweight. P22: COPD, Left foot nerve disorder. P23: None. P24: Hypertension, glaucoma, iron deficiency anemia. P25: none. P26: Obesity, Schatzki ring in esophagus, migraines, myofascial syndrome arms. P27: Congestive hepatopathy secondary to Fontan associated liver disease with established cirrhosis. Double inlet left ventricle with straddling of the right AV valve. Double outlet right ventricle with mild subaortic stenosis. Bilateral superior vena cava with left SVC draining into the coronary sinus. Non-restricted VSD. Fontan procedure in 1989. Cryoablation of WPW posterior accessory pathway. Atrial arrhythmias with interatrial reentrant tachycardias, atypical atrial flutter, sinus node dysfunction, and AV node dysfunction. Required epicardial dual chamber pacemaker 2018. Mixed pattern lung disease. Tracheostomy at the time of initial cardiac surgery. Strangulated umbilical hernia with infarcted small bowel requiring surgical repair 2019. Remote meningitis. Complex congential heart disease with failed Fontan circulation and single ventricle physiology. Cardiac cirrhosis complicated by ascites and stage 3b-4 CKD. Cumulative PRA 98%. Previous tobacco use, currently no smoking no EtOH. Remdesivir (D30). |
| Lu 2020 | After discharge, 77/87 re-positive cases were asymptomatic, and 10 had a symptom of unproductive cough, mainly at night. All repositive cases had only mild (46) or moderate (41) clinical symptoms during initial hospitalization. | Clinical data were obtained through Guangdong’s COVID-19 surveillance network | Hospitalized for COVID-19 before quarantine (all had mild/moderate COVID-19). Chronic conditions: N/R |
| Mancon 2022 | Fever, headache, cough. Pneumonia. | N/R | Folicular lymphoma successfully treated with obinutuzumab plus cyclophosphamide, doxorubicin, vincristine, prednisone (G-CHOP). D38 - started ciprofloxacin (urinary tract infection), switched to amoxicillin/clavulanic acid (E. feacalis isolation). D50 - voriconazole plus piperacillin /tazobactam regimen was started. D323- Cominarty vaccine |
| Mendes-Correa 2021 | D0 - fever (37.8°C), myalgia and headache. Severe symptoms (ICU on hospitalization 2). | N/R | Hematopoietic stem cell transplantation (HSCT) to treat a diffuse large B-cell lymphoma. At six months after HSCT the patient was on maintenance therapy with acyclovir, sulfamethoxazole, trimethoprim, glucocorticoids and cyclophosphamide. During hospitalization 2 - several bacterial infections related to this prolonged hospitalization. BAL identified a ventilator pneumonia due to Klebsiella sp and Stenotrophomonas maltophilia. Convalescent plasma. Hospitalization 3 - convalescent plasma. |
| Mileto 2021 | Dyspnoea and dry cough. | N/R | COPD, valvulopathy, atrial fibrillation (treated with acenocoumarol) and prostatic cancer (treated with bicalutamide); he had been vaccinated against influenza. Lopinavir/ritonavir and hydroxychloroquine. Ceftriaxone (7 days) and azithromycin (5 days). |
| Monrad 2021 | Asymptomatic D0-D11, D101-D333. Symptomatic D12-D101. Respiratory symptoms, fever. | Data were obtained from electronic patient records. | Remdesivir D24-D33, D60-D65. Convalescent plasma D70, D126, D127. Ibrutinib to D24. |
| Murata 2021 | Asymptomatic | Asymptomatic status was determined at the time of testing based on the absence of fever (temperature ≥37.5°C) and clinical symptoms (cough, dyspnea, chest pain, sore throat, and nasal discharge) by physicians and nurses. The body temperature, oxygen saturation, and symptoms were monitored at least twice a day. | Carrier_1: diabetes and hypertension |
| Nissen 2021 | D- days from first RT-PCR positive. D3 - temperature of 38°C, nasal congestion and mild sinusitis symptoms. By D10 , the fever had subsided, but the nasal congestion continued until D7. On D8 she had fully recovered. | N/R | No comorbidities |
| Niyonkuru 2021 | Patient 1: Symptoms starting D6. Patient 2: asymptomatic | N/R | P1: recent liver transplant. Mycophenolatmofetil 500 mg twice daily, tacrolimus 4 mg × 2 and prednisolone 15 mg × 1. D14 - mycophenolatmofetil was paused and tacrolimus was reduced in dose. D25-D29 - Remdesivir. P2 - multiple myeloma and previous autologous bone marrow transplantation, pacemaker. Lenalidomide 25 mg daily. |
| Nomura 2022 | Respiratory failure - mechanical ventilation | Patient information (date of symptom onset, medical history, medications, body mass index [BMI]) was extracted from the hospital’s electronic medical records. Medical history of patients was only included in this study when there was evidence of a link between having a medical history and the occurrence of severe COVID-19 | P1: Rheumatoid arthritis (receiving prednisolone and abatacept), end stage renal failure (undergoing dialysis), autoimmune leukopenia. Favipiravir D 3–10; corticosteroids D8–18. P2: Fatty liver. Favipiravir D3–8; corticosteroids D4–11; High-dose pulse therapy: 9 days. P3: Hypertension. Corticosteroids D4-13. P4: Hypertension. Corticosteroids D9–15. P5: Hypertension. Favipiravir: D4–5; Corticosteroids D4–17; High-dose pulse therapy: D4–6, D12–14. P6: Rheumatoid arthritis (receiving Janus kinase inhibitor and prednisolone), hypertension. Remdesivir: D3; corticosteroids D3–10 |
| Pedro 2021 | Fever, dry cough, pleuritic, chest pain, and vomiting; hypoxic to 88% on room air. After six days of inpatient care, she complained of left upper limb pain with signs consistent with deep vein thrombosis associated with indwelling peripheral venous catheter. D74-D76: nearly two months after discharge, she was re-admitted with headaches, fever, myalgia, and right pleuritic chest pain. Her vital signs were 110/52 mmHg blood pressure, 112 bpm heart rate, and 98.7 F body temperature. Her peripheral blood oxygen saturation was 97% on room air; all of the blood work values were within the normal range, non immunocompromised | D0-D9 - anamnesis. | No comorbidities |
| Pérez-Lago 2021 | Case A - symptomatic; Case B - symptomatic; Case C- symptomatic. All were admitted to ICU. Respiratory symptoms, fever. | N/R | **Case A** - Follicular lymphoma in remission; rituximab treatment every 3 months. Lopinavir/ritonavir D66-75, D85-99, D133-194. Remdesivir D75-84, D98-107, D126-133. Hyperimmune plasma D127-128. **Case B** - Follicular lymphoma treated with rituximab-bendamustine. Lopinavir/ritonavir D16-27, D54-63, D67-76. Remdesivir D68-77, D82-91. Hyperimmune plasma D69, D82. **Case C** - Ellis-Van-Creveld syndrome, grade IV follicular lymphoma in complete remission. Persistent lymphopenia. Lopinavir/ritonavir D0-14, D16-69. Remdesivir D32-42, D49-58. Hyperimmune plasma D58. |
| Pickering 2021 | Patient 1, patient 2 - asymptomatic. Patient 3 - severe COVID-19. Patient 4, patent 5 - mild COVID-19 | N/R | P1: Hypertension, pancreatic cancer. P2: Hypertension, IgA nephropathy, ischemic heart disease. P3: End-stage renal failure, renal transplant, obesity, type-2 diabetes. P4: Immune thrombocytopenia. P5: Rectal cancer. |
| Rajakumar 2021 | Case 1: Nasal stuffiness and discharge, sneezing, fatigue, and cough (D0). Respiratory symptoms. Case 2: progressive dyspnoea and hypoxemia requiring intubation. | See Lin 2022 | **Case 1:** Recent cardiac transplant (antithymocyte globulin induction and standard triple immunosuppressive therapy). Prednisone, tacrolimus, mycophenolate mofetil, and standard prophylactic medications. Type-2 diabetes mellitus, hypothyroidism, osteoporosis, anaemia. Remdesivir D27-D36. **Case 2:** Recent cardiac transplant (prednisone, tacrolimus, MMF, and standard prophylactic medications). Acute graft rejection. Liver cirrhosis, kidney disease. Remdesivir (D25-D30) |
| Sepulcri 2021 | Fever, dry cough, and respiratory distress (O2 supply) | N/R | Non-Hodgkin lymphoma (2 cycles of rituximab, bendamustine, cytarabine). Arterial hypertension, atrial flutter, benign prostatic hypertrophy, hypercholesterolemia. B-cell depletion, hypo-IgG and -IgM. CMV infection (treated with ganciclovir). Darunavir/ritonavir, hydroxychloroquine, immunoglobulins (D38-40, D96-98, D170-172, D189-191), Remdesivir (D47-51, D77-86, D178-182, D205-209), convalescent plasma (D88), steroids, tocilizumab (D32-33, D86). |
| Siedner 2022 | All delta variant infections in the cohort were mild, but symptomatic. | Symptoms were assessed at each specimen collection and through medical chart review after study completion. Symptomatic infections were defined as those with COVID-19–related symptoms at any point during the observation period. | Vaccination |
| Singh 2021 | All patients had respiratory symptoms, characterized by cough, congestion, and/or dyspnoea. Thirteen patients exhibited findings on chest radiography consistent with a COVID-19 diagnosis. Four patients had at least a one-night stay in the ICU (Patients 2, 5, 10, 12). | N/R | ≥ 1 chronic condition. Ten patients were chronically bedbound (Patients A5-A14). |
| Sung 2022 | Case 1. Symptomatic. Severe COVID-10. Case 2: symptomatic (fever, shortness of breath, cough). Case 3-12 - unclear | Chart review. | P1: chronic lymphocytic leukaemia undergoing treatment with venetoclax and obinutuzumab. Chronic kidney disease. Fibromyalgia. Hyperlipidaemia. P2: Marginal zone lymphoma treated with bendamustine, rituximab. Hyperlipidaemia. Deep vein thrombosis. Acute haemolytic anaemia. |
| Tarhini 2021 | Patient 1: symptomatic (confusion, respiratory signs on CT). Patient 2: symptomatic from D0 - (asthenia, dry cough, myalgia, and low-grade fever). On D76 - dry cough, dyspnoea, and oxygen requirement. Patient 3: from D0 - fever, cough, mild dyspnoea. D49 - persistent cough, exertional dyspnoea, and intermittent fever. D73 - the patient had recurrence of fever, cough. | N/R | P1: HIV and PML. P2: Heart transplant (prednisone, mycophenolic acid, belatacept). Diabetes mellitus. Chronic kidney disease. Obstructive sleep apnoea, gout disease, osteoarthritis, arterial hypertension. P3: Rheumatoid arthritis under treatment with rituximab. Lymphopenia. |
| Thornton 2022 | Respiratory symptoms, fever. | See Lin 2022 | Follicular lymphoma in remission. Lymphopenia. Hypogammaglobinaemia. Remdesivir D90-94, D118-122, D146-155. Bamlanivimab D133. IVIG D107, D137, D165, D193 |
| Truong 2021 | **Patient 1:** pre-symptomatic (D0). D3- fatigue, vomiting , cough, malaise, and gastrointestinal symptoms. Other symptoms: sore throat. **Patient 2:** pre-symptomatic (D0). D4 - cough, D6 - decreased appetite, fever. Other symptoms: malaise, shortness of breath, congestion/rhinorrhoea, vomiting, abdominal pain. **Patient 3:** symptomatic from D0. Other symptoms: fever, malaise, cough, sore throat, shortness of breath, congestion/rhinorrhoea, vomiting, abdominal pain. | N/R | P1: child with B-cell acute lymphoblastic leukaemia (Chemotherapy per COG AALL0932: dexamethasone, vincristine, peg-asparaginase, methotrexate, 6-MP, doxorubicin). P2**:** adult with B-cell acute lymphoblastic leukaemia (Cyclophosphamide and Fludarabine as lymphodepleting agents prior to CAR-T cell therapy). Prolonged pancytopenia. Remdesivir (D18-22, D40-44). Weekly convalescent plasma therapy (D103- D144) and on D159. P3: child with B-cell acute lymphoblastic leukaemia (HR B-ALL chemotherapy per COG AALL0232: Cyclophosphamide, cytarabine, thioguanine, vincristine, dexamethasone, methotrexate, mercaptopurine). Remdesivir (D32-D36). |
| Weigang 2021 | No respiratory symptoms at the time of diagnosis. The CT scan showed mild ground-glass opacities and discrete bilateral pleural effusions on D4. During the 25 weeks of infection with SARS-CoV-2 the patient had no severe respiratory or systemic symptoms. | N/R | Immunosuppressed patient with the first positive RT-qPCR positive result 12 days after kidney transplantation. Renal transplant (for autosomal dominant polycystic kidney disease); tacrolimus, mycophenolate, prednisone. Basiliximab (20mg, day 0 and day 4 post-transplantation). Coronary heart disease, arterial hypertension, hyperlipidaemia, obesity. Ivermectin (D56-D60). Urinary tract infection with E. coli. Kidney transplant failure (D106). Remdesivir D140-149. |
| Williamson 2021 | Respiratory symptoms. | N/R | Chronic lymphocytic leukaemia (six cycles of fludarabine /cyclophosphamide /rituximab). Hypogammaglobulinaemia. Neutropenia. Lymphopenia. Azithromycin. Remdesivir (D222-238), intravenous human immunoglobulin (D225). Casirivimab and imdevimab (D265-). |
| Zahn 2021 | Patient 1, Patient 2 - symptomatic (mild COVID-19). Patient 3 - asymptomatic | N/R | P1: Hypothyroidism. P2: Migraine. P3: None |
| Zupin 2022 | Pre-symptomatic. Fever, respiratory symptoms (D4 - D42) | N/R | Comorbidities - N/R. Azithromycin D7-D12. Prednisone D23-D30 |
| Garcia-Knight 2022 | In SR: symptomatic 50 unvaccinated, 30 vaccinated. Pre-symptomatic: 1 unvaccinated, 1 vaccinated. | Symptom surveys, clinical and epidemiologic questionnaires, done either by phone or written, completed on dE, and on days 9, 14, 21, and 28 after index case symptom onset. | 82: no immunosuppression |
| Kang 2023 | Symptomatic 82 (vaccinated with 1st booster = 75). All the participants, even those who were initially asymptomatic (n=15), eventually developed symptoms during the isolation period | Questionnaire sheet. All participants were instructed to record daily symptoms based on a 5-score scale (asymptomatic: 0, mild: 1, moderate: 2, substantial: 3, and severe: 4) that comprised 24 symptoms | Vaccinated with 1st booster: 75. Days from last vaccination (mean ± SD) 184.7 ± 71.4 |
| Kim 2022b | Symptomatic. Severe or critical COVID-19 | Clinical and epidemiologic metadata were obtained through patient or family interview, electronic medical record review, and the Illinois’ National Electronic Disease Surveillance System to confirm SARS-CoV-2 test results and date of symptom onset for events outside the study sites. | Not vaccinated. P1: Non-Hodgkin lymphoma (maintenance with rituximab). Kidney transplant (sirolimus and prednisone 2.5 mg daily). Remdesivir, dexamethasone. P2: CLL with Richter’s transformation s/p autologous stem cell transplant on maintenance ibrutinib. Remdesivir, dexamethasone.P3: Kidney transplant on mycophenolate mofetil, tacrolimus, and prednisone 10 mg daily. Remdesivir, dexamethasone. P4: Gastric MALT lymphoma and multiple sclerosis on maintenance ofatumumab. Hypogammaglobulinemia. Remdesivir, dexamethasone. P5: Remdesivir, dexamethasone. |
| Luna-Muschi 2022 | Mild COVID-19. Symptomatic. | Clinical data were obtained at baseline, and symptom duration was monitored by telephone up to resolution of symptoms or 14 days after symptom onset, whichever was later. | All participants had been immunized with at least 2 doses of any COVID-19 vaccine. Previous COVID-19: 10/30 |
| McCormick 2023 | Pre-symptomatic: 3. Symptomatic: 3. Asymptomatic: 3. Not available: 2. | The authors collected and managed demographic and symptom information using the Research Electronic Data Capture (REDCap). Information on symptoms available for 10/12 participants. Asymptomatic: 4 | 10/12 participants (83%) had completed a primary COVID-19 vaccination series prior to the outbreak with either Pfizer/ BNT162b2 (4/10, 40%), Moderna/mRNA-1273 (3/10, 30%), or Janssen/JNJ-78436735 (3/10, 30%). 2 vaccinated persons had a documented prior SARS-CoV-2 infection (both > 6 months prior to current illness onset). Both of these persons received their COVID-19 vaccination series following infection with SARS-CoV-2. 11/12 participants had at least one underlying medical condition, the most common being overweight with BMI > 25 (10/12, 83% including 5/12, 42% with BMI > 30) and history of tobacco use disorder (7/12, 58%). |
| Tobolowski 2022 | 9/11 symptomatic, 2/11 asymptomatic 7/11 with mild to moderate disease (mild to moderate includes also asymptomatic) and 4/11 severe disease with following definitions 1. Mild illness: asymptomatic COVID-19 + those who reported COVID-19 signs or symptoms without dyspnoea or evidence of lower respiratory tract involvement.  2, Moderate illness included participants with signs of lower respiratory disease by clinical assessment or imaging,  3. severe disease included participants with tachypnoea (>30 breaths per minute), oxygen saturation <90% (or decrease from baseline over 3% for at least two consecutive values), hospitalization, or death | Electronic medical chart abstraction was performed to obtain supplemental information on participants’ past medical history, medications, laboratory test results, clinical outcomes, and vaccine administration. Information from questionnaires and chart abstraction were entered into a Research Electronic Data Capture (REDCap) database. | Median age [range]: 74 [37–90]. Most common underlying conditions: cardiovascular disease (11, 100%) and diabetes (6, 55%); 10 (91%) participants had >3 underlying conditions. One participant (A) was immunocompromised with B-cell lymphoma. |
| Spinicci 2022 | middle March 2020: fever, dry cough, anosmia, and headache soon after a household contact with a confirmed COVID-19 case | INFECTION: confirmatory test not performed - being classified as a probable COVID-19 case; the patient recovered spontaneously in a few days and resumed his life without limitations All other data were obtained from electronic health records | Eosinophilic granulomatosis with polyangiitis (EGPA). Methylprednisolone (0.15 mg/kg/day), mycophenolate mofetil (1000 mg per day), rituximab. Asthma. Therapies for COVID-19: Mycophenolate mofetil (D0-D101). Convalescent plasma (D101 and 172). Remdesivir (D167–172). Bamlanivimab 700 mg and etesevimab 1400 mg (D231) |
| Choi 2020 | Chills, muscle aches and headache three days prior to hospitalization and subsequently developed dizziness, nausea without emesis, mid-central abdominal pain, anorexia and persistent fevers. 3 recurrences | N/R | History of catastrophic antiphospholipid syndrome (APS), complicated by venous thromboemboli, pulmonary emboli, thrombotic microangiopathy, adrenal hemorrhage, coronary vasculitis, and aortitis. Five months prior to presentation, he had been treated for diffuse alveolar hemorrhage (DAH). He was on warfarin for therapeutic anticoagulation, hydroxychloroquine, cyclophosphamide, and corticosteroids at prednisone 15 mg daily, and he had recently received rituximab and eculizumab. Remdesivir. Immunoglobulin,  cyclophosphamide, and daily ruxolitinib, in addition to glucocorticoids. Regeneron. |

Notes: N/R: not reported.
