# Supplementary material for: Serial cycle threshold to assess the infectious potential of SARS-CoV-2: A systematic review
Source: Epidemiol Infect. 2026 May 6;154:e89. doi: 10.1017/S0950268826101484 (PMC13366375; doi:10.1017/S0950268826101484)
Supplement: Rosca et al. supplementary material [file S0950268826101484sup001.zip › WebTable 3 and WebFigure 1.docx]

WebTable 3: Descriptive analysis of Ct values for which viral cultures were completed

| Group | Viral Culture Results | N (Ct values)† | Median Ct | IQR Ct |
| --- | --- | --- | --- | --- |
| 1 | Positive cultures | 336 | 19.5 | 6.1 |
| 2 | Negative cultures | 1057 | 32.8 | 11.0 |

Legend: All numeric Ct (Cycle threshold) values from each patient for whom viral cultures had been completed and reported as positive or negative growth were extracted and the median, and IQR (interquartile range) with and without outliers were calculated. For the group of positive cultures "without outliers,” (n=322) any value beyond the standard 5×IQR rule from the 25th or 75th percentile was excluded before re-computing the metrics. Removing the outliers from the positive culture group resulted in a lower mean Ct value of 19.8, a lower median of 19.0 and a lower IQR Ct value of 5.9

WebFigure 1: Visualizations of the data distributions for positive and negative viral cultures versus Ct value using a distribution histogram, boxplots, Q-Q (quantile-quantile) plots and Receiver Operating Characteristic (ROC) curve analyses for Ct value thresholds


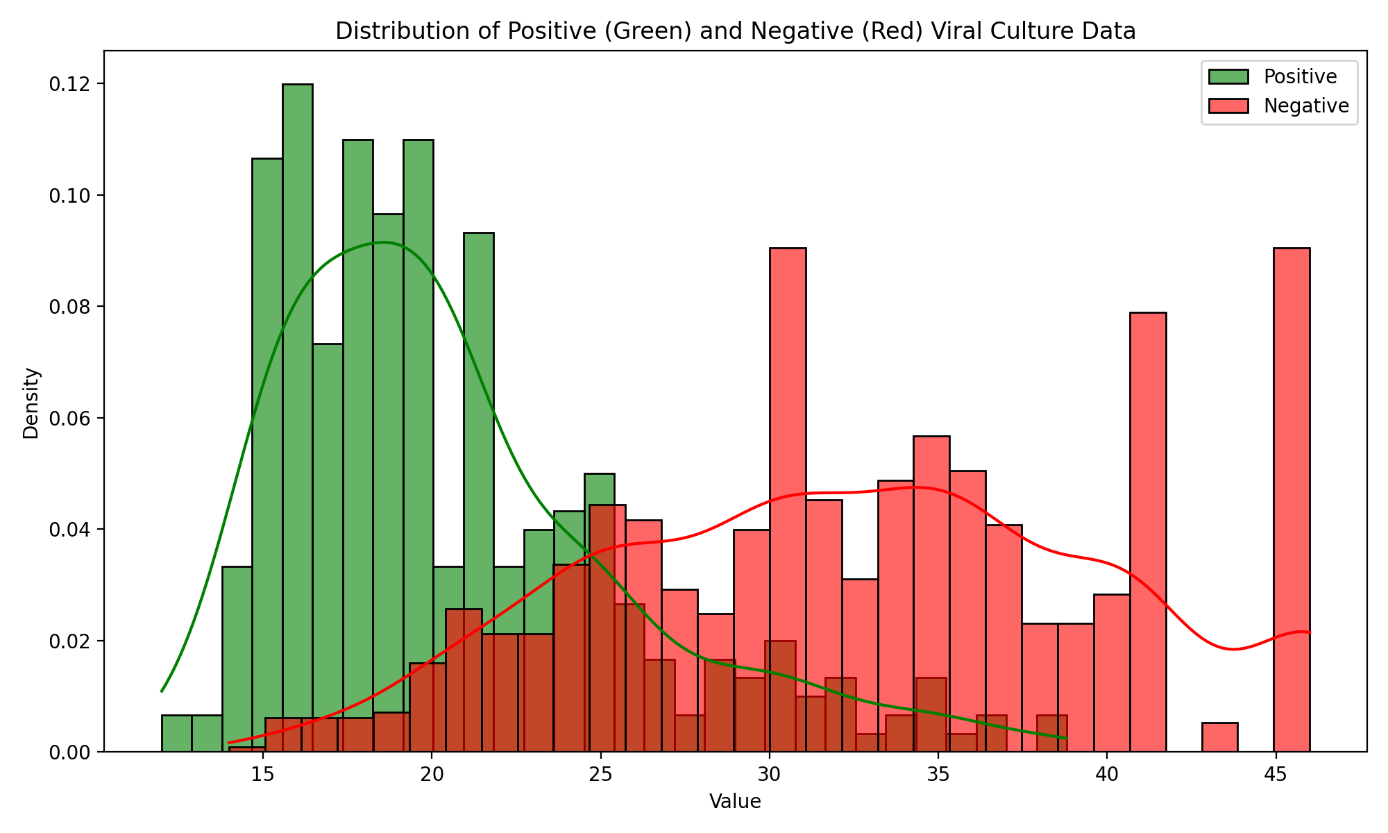


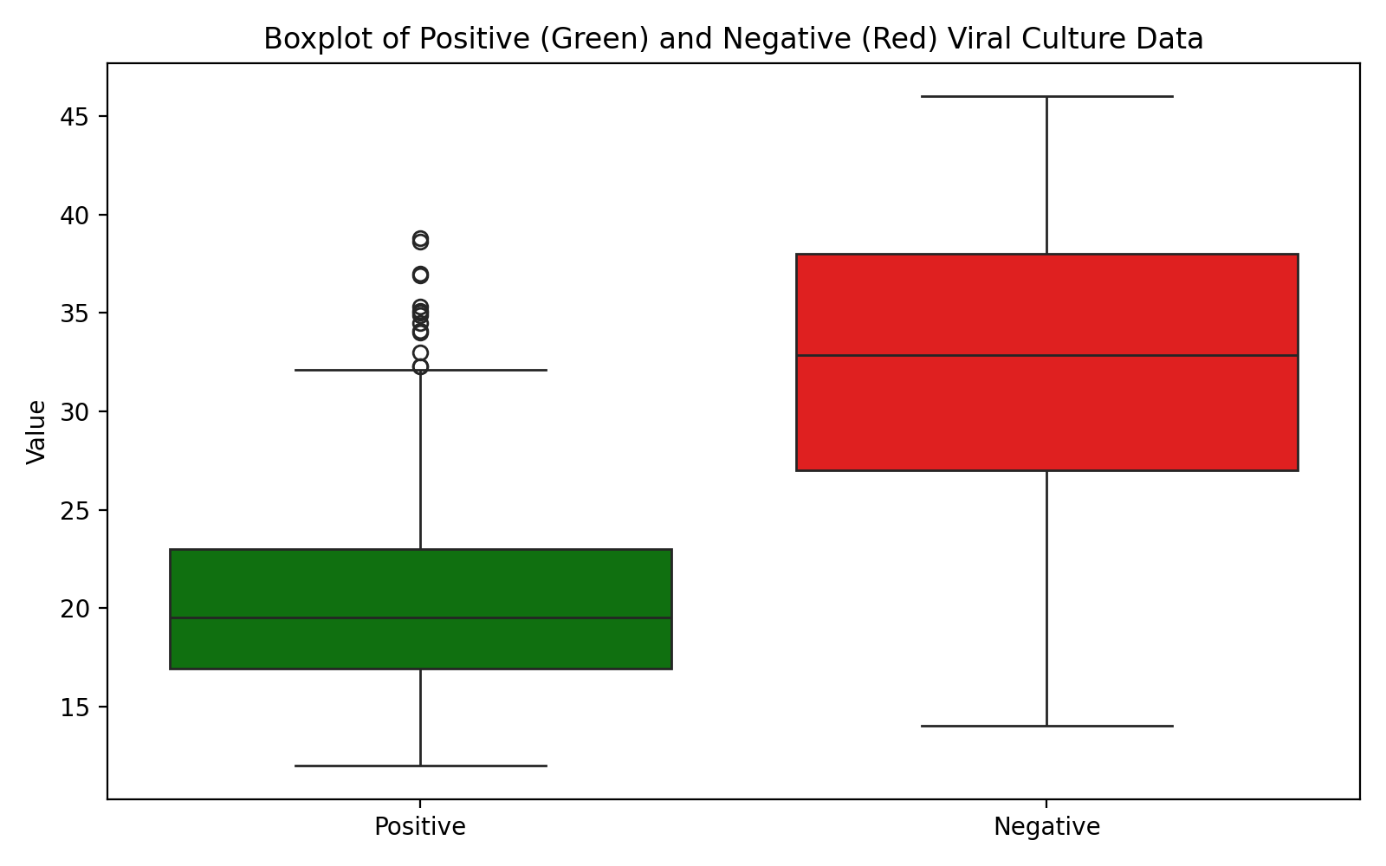


.

Q-Q plots for the culture positive and negative groups*


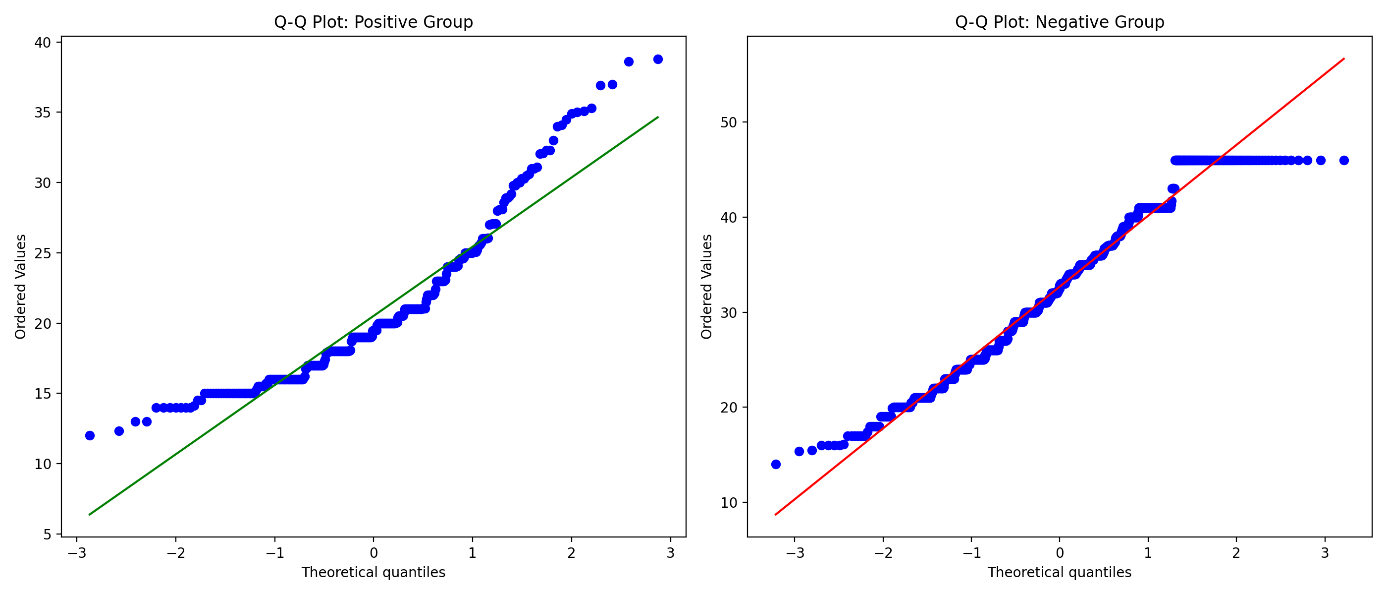


*The Q-Q plot demonsrates the data do not follow a normal distribution with deviations at extreme values in both directions. Comparison of Ct Values between the positive and negative culture groups, with and without outliers, in the culture positive and negative groups using a Mann Whitney U test revealed a highly significant difference in Ct values p<0.0001)

Receiver operating characteristic (ROC) curve analyses for Ct value thresholds with and without outliers


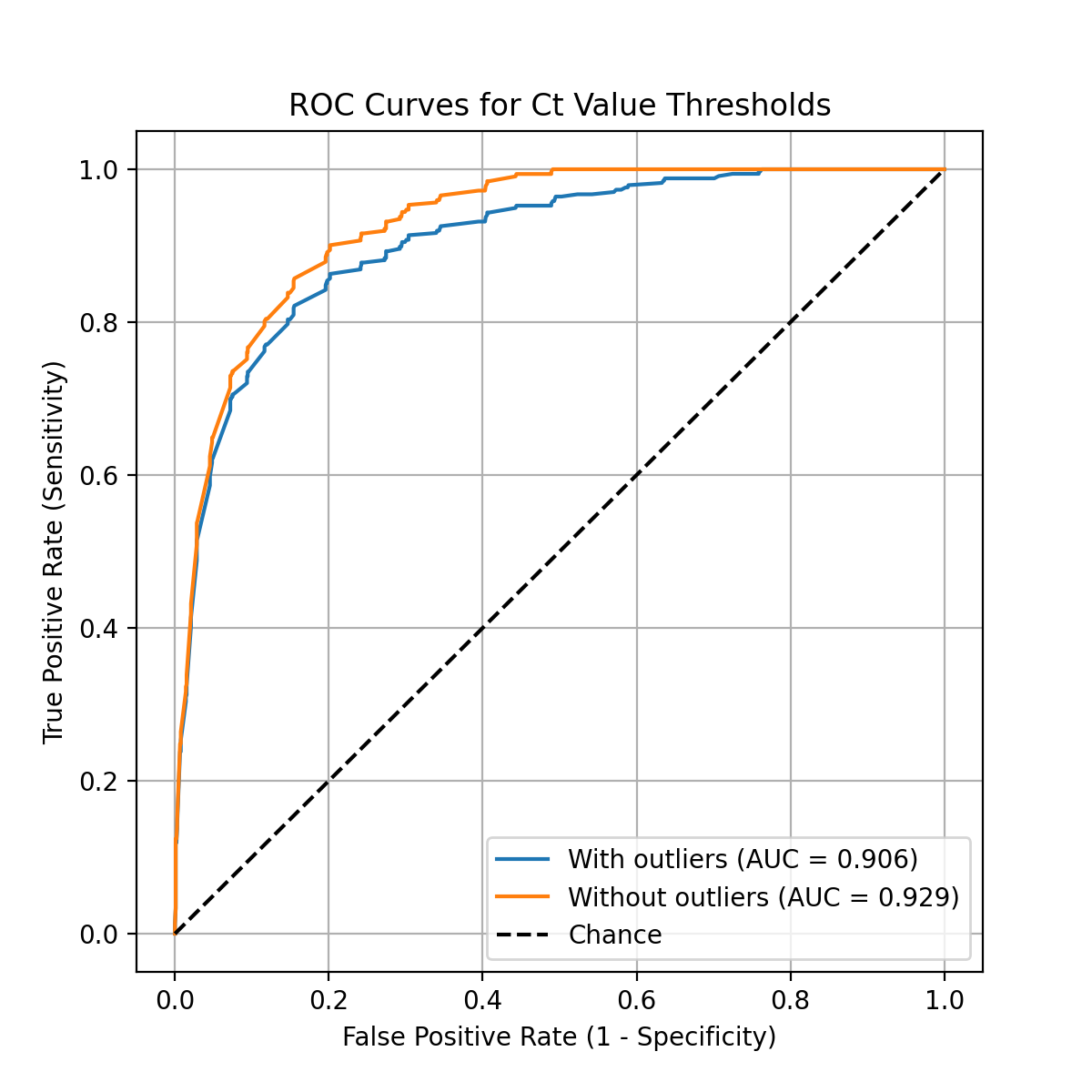


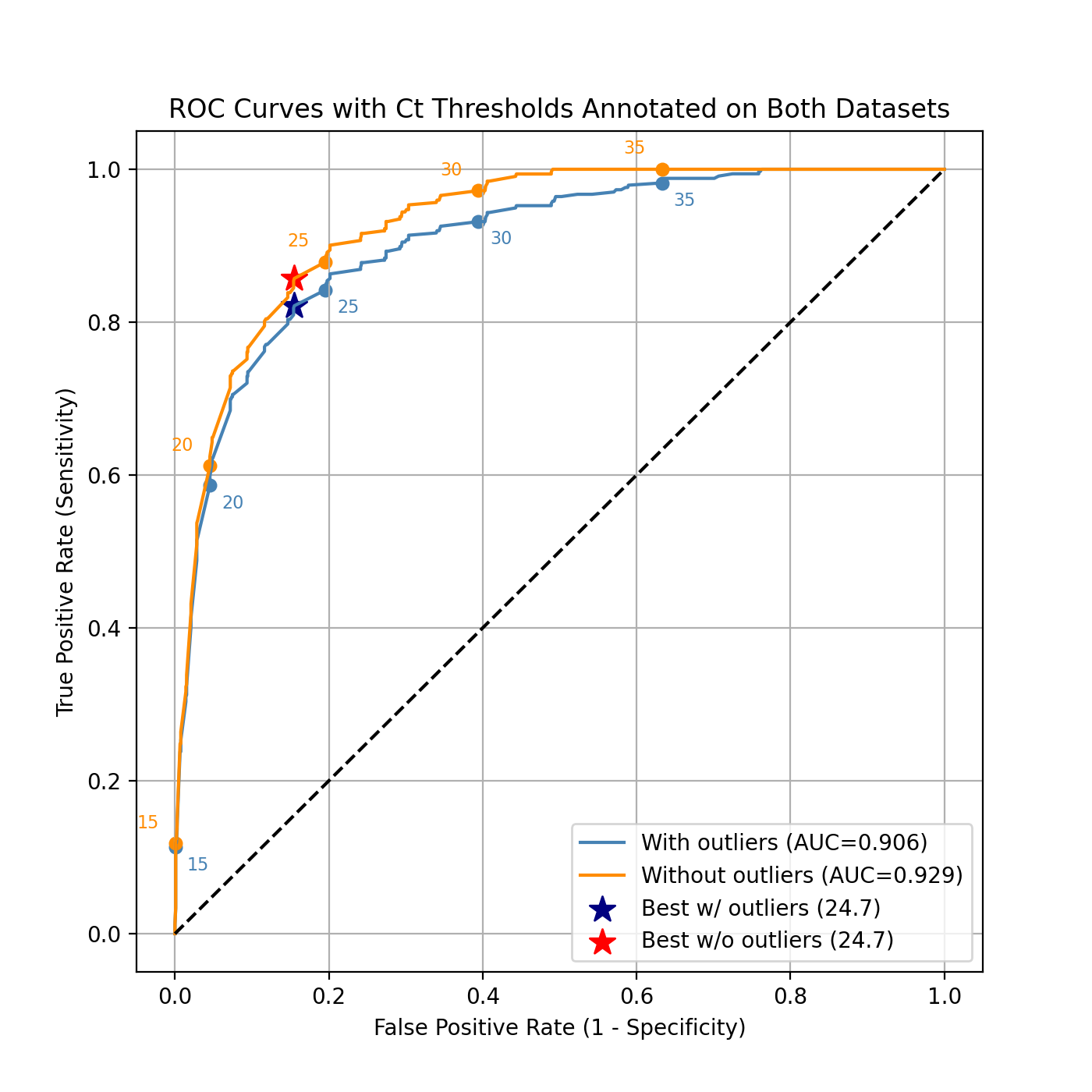
 Legend: Both curves confirm that Ct is a strong predictor of viral culturability and demonstrates using outlier-cleaned data yields a small but meaningful boost in sensitivity at any fixed specificity—and the optimal cut-point (≈ 24.7 cycles) remains unchanged. Navy star ≈ 24.7 cycles (raw data with outliers) Red star ≈ 24.7 cycles (cleaned data). Removing outliers lifts the orange curve consistently above the blue, confirming a gain in performance at nearly every threshold. The optimal cut-off remains identical (≈ 24.7 Ct) in both analyses, underscoring its robustness. At that point, sensitivity improves from 82% to 86%, while specificity remains steady (~85%). The benefit is most evident in the Ct 25–30 region, where there is the greatest clinical debate but our analysis with outlier removal reveals several percentage points of sensitivity gain without sacrificing specificity. Lower Ct cut-offs (15–20) already perform nearly perfectly; they change little, confirming that outliers mainly affect mid-range and higher thresholds.
