# Supplementary material for: Serial cycle threshold to assess the infectious potential of SARS-CoV-2: A systematic review
Source: Epidemiol Infect. 2026 May 6;154:e89. doi: 10.1017/S0950268826101484 (PMC13366375; doi:10.1017/S0950268826101484)
Supplement: Rosca et al. supplementary material [file S0950268826101484sup001.zip › WebTable 4. Univariate associations.docx]

### **WebTable 4.** Univariate associations with cycle threshold value (based on first RT-PCR test values per patient)

| **Characteristic** | **Number of patients**  **(number of studies)** | **Difference in Ct from reference level (95% CI)**  *First row per each category mean Ct (reference level)* | **P value** |
| --- | --- | --- | --- |
| ***Culture Positive*** | | | |
| No | N = 233 (k = 21) | 29.3 (27.3 to 31.1) | - |
| Yes | N = 148 (34) | -7.63 (-8.96, -6.32) | P < 0.001 |
| ***Time time since first PCR*** | | | |
| < 7 days | N = 183 (k = 17) | 21.5 (19.3 to 23.9) | - |
| 7 - 13 days | N = 67 (16) | +3.19 (1.15 to 5.21) | P < 0.001 |
| 14 - 27 days | N = 99 (16) | +5.86 (3.54 to 8.20) | P < 0.001 |
| +28 days | N = 16 (16) | +7.08 (3.86 to 10.3) | P < 0.001 |
| ***Symptoms*** | | | |
| No data on symptoms reported | N = 47 (8) | 32.2 (29.2 to 35.2) | - |
| Pre/Asymptomatic | N = 85 (11) | -5.56 (-9.34 to -1.84) | P = 0.004 |
| Symptomatic | N = 249 (31) | -8.86 (-12.1 to -5.55) | P < 0.001 |
| ***Vaccination status*** | | | |
| Not data on vaccination status | N = 118 (16) | 25.7 (22.5 to 29.0) |  |
| No mention | N = 221 (21) | --0.68 ( -4.79, 3.30) | P = 0.749 |
| Yes | N = 42 (4) | -2.95 (-9.62, 3.75) | P = 0.392 |
| ***Platform*** | | | |
| N gene | N = 168 (15) | 25.5 (22.5 to 28.5) |  |
| E gene | N = 80 (14) | -1.80 (-5.53 to 1.96) | P = 0.362 |
| S gene | N = 45 (2) | 0.46 (-4.77 to to 5.74) | P = 0.859 |
| Other | N = 88 (10) | -0.198 (-8.68 to 8.37) | P = 0.953 |
| ***Hospitalised*** | | | |
| Not data on hospitalisation | N = 78 (4) | 27.4 (22.8 to 32.0) |  |
| Not mentioned | N = 165 (15) | -1.10 (-6.00 to 3.73) | P = 0.661 |
| Yes | N = 138 (29) | -3.43 (-8.14 to 1.29) | P = 0.156 |
| ***Intensive care*** | | | |
| Not recorded | N = 135 (11) | 25.8 (22.80 to 28.8) |  |
| No | N = 224 (28) | -1.41 (-4.72, 1.76) | P = 0.398 |
| Yes | N = 22 (9) | +1.03 (-2.51, 4.58) | P = 0.562 |
| ***Medical conditions*** | | | |
| No data on medical conditions | N = 199 (8) | 24.6 (22.4 to 26.8) |  |
| Data on medical conditions extracted from papers | N = 182 (35) | +2.05 (-1.58 to 5.63) | P = 0.25 |
| ***Cancer (inc solid and blood)**** | | | |
| No mention | N = 135 (20) | 26.0 (23.8 to 28.2) | - |
| Yes | N = 47 (24) | -3.39 (-5.87 to -0.842) | P = 0.009 |
| ***At least one chronic condition (inc diabetes, hypertension, IHD, etc)*** | | | |
| Not mentioned | N = 24 (6) | 27.2 (23.7 to 30.7) |  |
| Yes | N = 157 (31) | -3.23 (-6.44 to 0.050) | P = 0.054 |
| ***Immunocompromised (including on immune-suppressive drugs)*** | | | |
| Not mentioned | N = 133 (23) | 24.9 (22.5 to 27.3) |  |
| Yes | N = 49 (19) | -1.12 (-4.07 to 1.90) | P = 0.472 |
| ***Transplant*** | | | |
| No | N = 156 (29) | 25.2 (23.0 to 27.3) |  |
| Yes | N = 26 (11) | -3.31 (-6.41 to -0.129) | P = 0.042 |
| ***Covid-19 Treatments (Remdesivir, Dexamethasone,etc)*** | | | |
| Not mentioned | N = 158 (22) | 26.5 (24.3 to 28.70) |  |
| Yes | N = 24 (16) | -6.02 (-9.65 to -2.48) | P = 0.002 |
| ***Lineage*** | | | |
| No data on Lineage | N = 196 (20) | 26.2 (23.8 to 28.6) |  |
| A | N = 7 (5) | -5.36 (-10.70 to -0.07) | P = 0.047 |
| B | N = 103 (15) | -2.68 ( -5.67 to 0.27) | P = 0.075 |
| C/Delta | N = 45 (5) | + 0.80 ( -5.27 to 6.85) | P = 0.802 |
| Omicron | N = 30 (1) | -6.31 (-16.40 to 3.80) | P = 0.238 |
