# Supplementary material for: Serial cycle threshold to assess the infectious potential of SARS-CoV-2: A systematic review
Source: Epidemiol Infect. 2026 May 6;154:e89. doi: 10.1017/S0950268826101484 (PMC13366375; doi:10.1017/S0950268826101484)
Supplement: Rosca et al. supplementary material [file S0950268826101484sup001.zip › WebTable 5. Outliers.docx]

**WebTable 5.** Characteristics of culture-positive outliers

| **Study** | **Specimen** | **PCR platform / Gene** | **Culture system** | **Timing** | **Notes** |
| --- | --- | --- | --- | --- | --- |
| De Carvalho Leitão 2021 | NP swab | CDC assay | African green monkey cells | ≥ Day 14 | The samples from which viruses were isolated had multiple serial passages until a positive culture was obtained, with Ct values ranging from 15.0 to 38.8 being reported. |
| Gniazdowski 2021 | NP swab | RedStar (S gene) or NeuMoDx (NSP2) | Vero cells | Day 16 after first RT-PCR positive | No intervening samples from initial isolation until Day 16 and asymptomatic case.  CPE maintained up to 4 days. |
| Ke 2021 | NP swab | Abbott Alinity | Vero TMPRSS2 | ≥ Day 7 | Ct values of 3 cases with Ct values of 33, 34 and 35 reporting culture positive samples but with between 3-6 culture negative prior samples ranging from Day 7-11 and with between 1- 4 culture negative samples following amidst rising Ct values, raising the possibility of cell culture contamination |
| McCormick 2023 | NP swab | CDC assay | Vero TMPRSS2 | Day 5 | Asymptomatic case positive culture with Ct of 32.2 with negative samples at lower Cts. Only sample in the entire dataset culture + with a Ct > 30. Monitored for CPE until Day 7. |
